# Survivin inhibition ameliorates liver fibrosis by inducing hepatic stellate cell senescence and depleting hepatic macrophage population

**DOI:** 10.1002/ccs3.12015

**Published:** 2024-01-25

**Authors:** Sachin Sharma, Shaikh Maryam Ghufran, Mehreen Aftab, Chhagan Bihari, Sampa Ghose, Subhrajit Biswas

**Affiliations:** ^1^ Amity Institute of Molecular Medicine and Stem Cell Research (AIMMSCR) Amity University Noida Uttar Pradesh India; ^2^ Department of Medicine University of California San Francisco California USA; ^3^ Heersink School of Medicine University of Alabama Birmingham USA; ^4^ Division of Cellular and Molecular Oncology National Institute of Cancer Prevention and Research (NICPR) Noida Uttar Pradesh India; ^5^ Department of Pathology Institute of Liver and Biliary Sciences (ILBS) New Delhi India; ^6^ Department of Medical Oncology All India Institute of Medical Sciences (AIIMS) New Delhi India

**Keywords:** hepatic stellate cells, inhibitor of apoptosis (IAP), liver fibrosis, macrophage polarization, macrophage subtype, senescence, survivin (BIRC5), transforming growth factor‐β1 (TGF‐β1)

## Abstract

Persistent activation of hepatic stellate cells (HSCs) in the injured liver leads to the progression of liver injury from fibrosis to detrimental cirrhosis. In a previous study, we have shown that survivin protein is upregulated during the early activation of HSCs, which triggers the onset of liver fibrosis. However, the therapeutic potential of targeting survivin in a fully established fibrotic liver needs to be investigated. In this study, we chemically induced hepatic fibrosis in mice using carbon tetrachloride (CCl4) for 6 weeks, which was followed by treatment with a survivin suppressant (YM155). We also evaluated survivin expression in fibrotic human liver tissues, primary HSCs, and HSC cell line by histological analysis. αSMA^+^ HSCs in human and mice fibrotic liver tissues showed enhanced survivin expression, whereas the hepatocytes and quiescent (qHSCs) displayed minimal expression. Alternatively, activated M2 macrophage subtype induced survivin expression in HSCs through the TGF‐β‐TGF‐β receptor‐I/II signaling. Inhibition of survivin in HSCs promoted cell cycle arrest and senescence, which eventually suppressed their activation. In vivo, YM155 treatment increased the expression of cell senescence makers in HSCs around fibrotic septa such as p53, p21, and *β*‐galactosidase. YM155 treatment in vivo also reduced the hepatic macrophage population and inflammatory cytokine expression in the liver. In conclusion, downregulation of survivin in the fibrotic liver decreases HSC activation by inducing cellular senescence and modulating macrophage cytokine expression that collectively ameliorates liver fibrosis.

## Graphical abstract

Survivin inhibition induces senescence in hepatic stellate cells and modulates macrophage plasticity. Schematics showing Survivin expression induces with activation of HSCs and M2‐macrophage promotes HSCs activation through TGF‐β1 mediated survivin expression. Inhibition of survivin decreased fibrogenic activity of activated HSCs and induces cellular senescence. Decreased proinflammtory macrophages in liver promotes switch of pro‐inflammatory fibrotic microenvironment toward pro‐resolving, collectively ameliorates liver fibrosis.

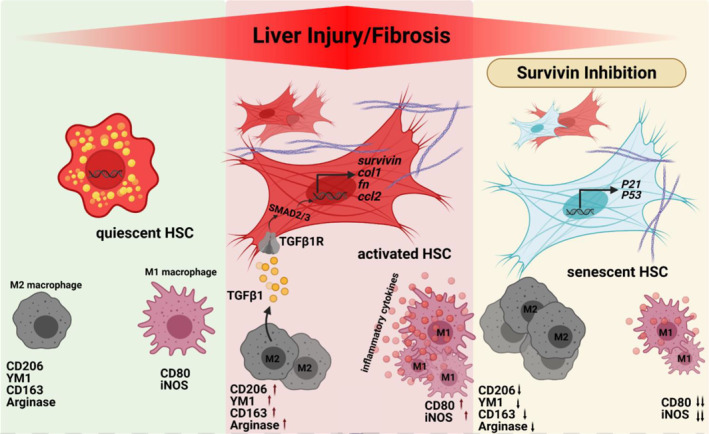

## 1 INTRODUCTION

Liver fibrosis is a common pathological wound healing response in various etiological end‐stage liver diseases and is characterized by the excessive accumulation of extracellular matrix (ECM) proteins.^1^ Hepatic stellate cells (HSCs) are the prominent players of the fibrotic response, located in the space of disse as a non‐fibrogenic, quiescent cell (qHSC) in a healthy liver.^2^ In the injured liver, HSCs become proliferative, fibrogenic, and activated (activated HSC) and show increased collagen I expression and deposition.^3^ The phenotypic conversion of HSCs involves vast changes in gene expression that drive the progression of liver fibrosis.^4^, ^5^

Survivin (BIRC5) is exclusively expressed in activated HSCs, and not in qHSCs.^5^, ^6^ It is a member of the inhibitor of apoptosis (IAP) protein family, predominately expressed in the proliferative cells but its expression decreases in terminally differentiated cells.^8^, ^9^ Functionally, survivin is considered an essential regulator of cell cycle and chromosomal segregation in association with Aurora B, INCENP, and Borealin that construct the chromosomal passenger complex (CPC).^10^ A low level of survivin expression has been reported in healthy and non‐neoplastic liver; however, it is upregulated in HCC.^11^ Survivin expression also increases during liver regeneration and is associated with hepatocyte cell division.^12^ Survivin knockout in hepatocytes showed an increased cell volume, macronuleation, and polyploidy with decreased proliferation without early induction of apoptosis. This suggests that survivin could be dispensable in the normal hepatocyte function.^13^, ^14^ Recently, we elucidated the role of survivin in the early activation of HSCs and liver fibrosis progression. We found that survivin expression in HSCs is essential for their activation that drives the onset of fibrosis following liver injury.^5^ However, the therapeutic potential of targeting survivin protein in activated HSCs at the fully developed liver fibrosis stage is largely unknown.

In the injured liver, macrophage phenotypic plasticity also regulates fibrosis progression.^15^ It has been reported that in the resolution phase of fibrosis, fibrogenic HSCs become inactivated and senescent or they are cleared by apoptosis.^16^, ^17^ Alternatively, activated M2 subtype macrophages secrete anti‐inflammatory cytokines that counterbalance inflammation and promote tissue repair.^18^

In this study, we inhibited survivin expression in activated HSCs and fibrotic mice livers using YM155 to understand the role of survivin in limiting the HSCs' fibrogenic response. We found that survivin expression was decreased in hepatocytes but increased in activated HSCs during chronic injury in the liver. Survivin suppression in fibrotic liver limits the activation of HSCs by inducing HSC senescence, and decreasing inflammatory cytokine gene expression that collectively ameliorates liver fibrosis.

## 2 RESULTS

### 2.1 HSC activation is associated with increased survivin expression

**FIGURE 1:**
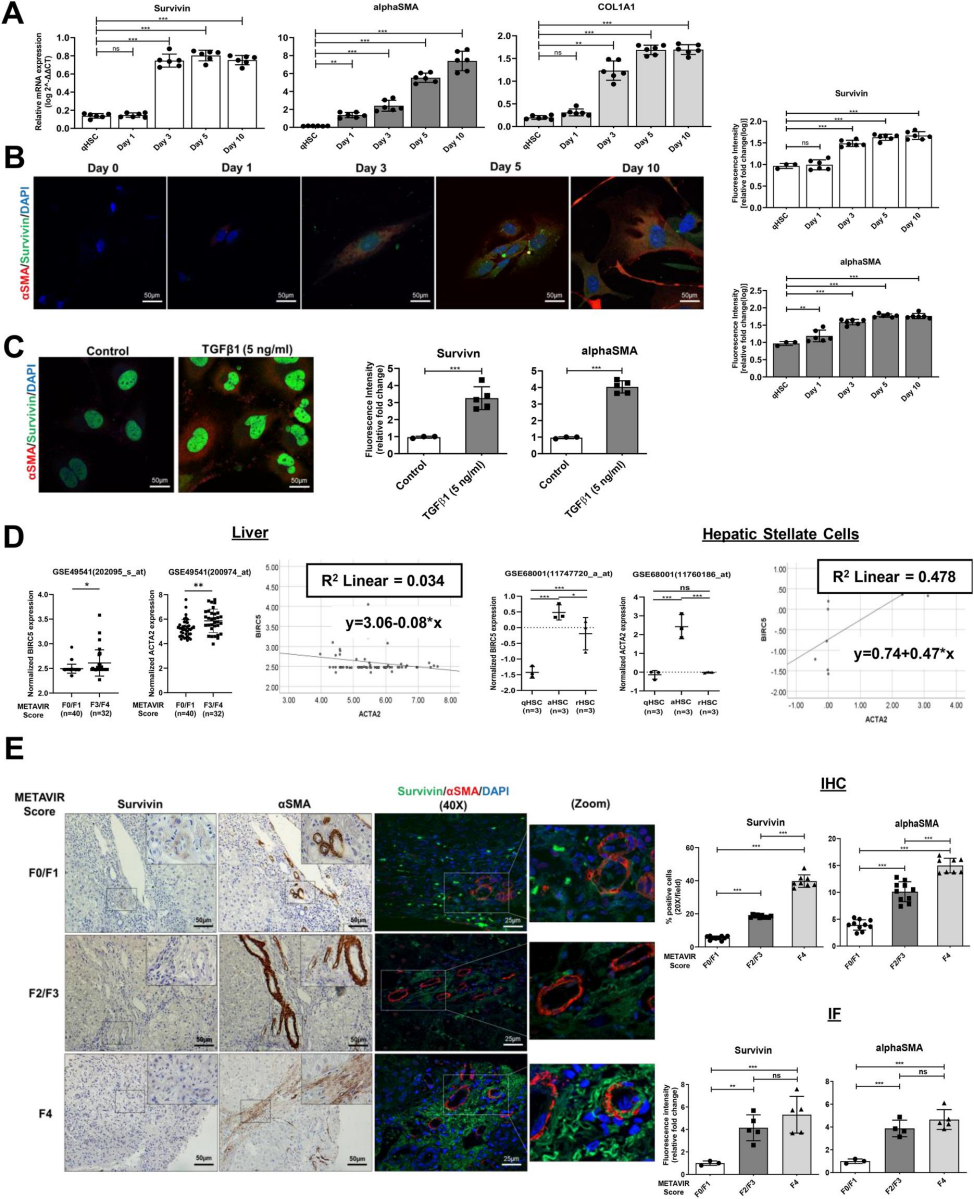
Survivin expression is upregulated in activated hepatic stellate cells. (A) mRNA expression of survivin, *α*‐SMA, COL1A1 in freshly isolated mice qHSCs, and activated HSCs at various time intervals. (B) Immunofluorescence (IF) images of freshly isolated mice qHSCs and activated HSCs at various time intervals with survivin (green), *α*‐SMA (red), and nucleus (blue) staining (scale bar: 50 μm; 63x magnification). (C) IF image of human HSCs, LX2 cells stimulated with TGF‐β1 (5 ng/mL) for 24 h demonstrating survivin (green), *α*‐SMA (red), and nucleus (blue) staining (scale bar: 50 μm; 63x magnification). (D) Survivin and *α*‐SMA expression in etiology independent F0/F1 METAVIR score liver (*n* = 40) and F3/F4 METAVIR score liver (*n* = 32); in quiescent HSC (qHSC), activated HSC (aHSC) and reverted HSC (rHSC) (*n* = 3 per group) from publicly available datasets. (E) Immunohistochemistry (IHC) and IF images of F0/F1 METAVIR score (*n* = 10), F2/F3 METAVIR score (*n* = 10), and F4 METAVIR score (*n* = 8) in etiology independent fibrotic grade human liver biopsy samples representing survivin (green), *α*‐SMA (red), and nucleus (blue) staining (scale bar: 50 μm; 20x and 63x magnification). All mice experiments were carried out with minimum *n* = 3 mice with two replicates each. nsP > 0.05, *P < 0.05, **P < 0.01, ***P < 0.001 [student's *t*‐test for (C, D); one‐way ANOVA for (E); two‐way ANOVA for (A, B, D)].

To determine the expression of survivin in HSCs, we selectively isolated primary qHSCs from healthy mice livers through liver perfusion and subjected them to flow cytometry‐based cell sorting (FACS) (Supplementary Figures S1A and S1B). The mouse primary qHSC was characterized by the retention of oil red stain in the cytoplasm, a hallmark of qHSCs (Supplementary Figure S1C). We examined the expression of survivin at different time intervals in activated HSCs through quantitative PCR (qPCR). While the HSC activation marker *α*‐SMA was induced after 24 h (D1) of culture, survivin and collagen I (COL1A1) expression were found significantly elevated at day 3 (D3) of HSC culture and from there onward to maximum expression at D10 (Figure 1A). Immunofluorescence (IF) microscopy further confirmed the increase in survivin protein expression at D3 and *α*‐SMA at D1 of primary HSC culture. However, freshly isolated qHSCs neither expressed survivin nor *α*‐SMA (Figure 1B), suggesting that the activation of HSCs from their quiescent state is associated with increased survivin expression.

Next, we stimulated the human HSC cell line, LX2 cells with the pro‐fibrogenic cytokine, TGF‐β1 (5 ng/mL).^19^ IF results showed a surge in survivin and *α*‐SMA protein expression in TGFβ‐1 stimulated LX2 cells compared to unstimulated control. As LX2 cells are activated HSCs or myofibroblast cells, a basal level of survivin expression was observed in unstimulated LX2 cells, which was however comparatively lower than that in TGFβ‐1 stimulated LX2 cells (Figure 1C). These results highlight that TGF‐β1 fibrogenic cytokine enhances survivin expression in activated HSCs.

We also evaluated survivin gene expression in a publicly available database for liver tissues from patients with different METAVIR scores. Survivin expression was found upregulated in liver tissue samples from patients with advanced fibrosis/cirrhosis stage (F3/F4 METAVIR score) compared to non‐fibrotic/mild fibrotic liver tissue samples (F0/F1 METAVIR score) (*p* < 0.05). *α*‐SMA expression was found to increase along increasing fibrosis grades; however, a positive correlation was not observed between *α*‐SMA and survivin expression (*R* ^2^ = 0.034) in fibrotic liver tissue samples. Interestingly, a database including expression profiles from qHSCs as well as reverted HSCs (rHSCs) isolated from resolved fibrotic livers showed low survivin expression. Activated HSCs displayed high survivin expression and also showed a positive correlation with *α*‐SMA expression (*R* ^2^ = 0.478) (Figure 1D). These results suggest that survivin expression is confined to activated HSCs. A positive correlation was not observed between survivin and *α*‐SMA expression from whole liver datasets possibly due to the presence of more hepatocytes and fewer survivin expression‐ activated HSCs in the injured liver. To confirm our results, we examined survivin expression by immunohistochemistry (IHC) in human liver biopsy samples from chronic liver disease patients with different fibrosis grades (METAVIR score). IHC results showed that survivin and *α*‐SMA expression was confined to the portal region in the non‐fibrotic/mild fibrotic liver tissue sections (F0/F1 METAVIR score) and extended to periportal regions in advanced fibrotic liver tissue sections (F2/F3 METAVIR score). However, in cirrhotic liver sections (F4 METAVIR score) survivin and *α*‐SMA expression were found throughout the fibrotic septa. IF studies confirmed the co‐localized expression of survivin and *α*‐SMA in the fibrotic area of human liver biopsy sections (Figure 1E). These results suggest that survivin expression is primarily upregulated in activated HSCs and it is negligible in qHSCs and hepatocytes in injured liver.

### 2.2 Survivin inhibition decreases the fibrogenic activity of activated HSCs

To investigate the effect of survivin inhibition on activated HSCs, we evaluated the cytotoxicity of YM155 on LX2 cells by MTT assay. YM155 at a concentration of 37.45 ± 1.15 nM decreased 50% of LX2 cell viability after 24 h of treatment (Supplementary Figure S2A). We also performed annexin–propidium iodide (PI) flow cytometry‐based apoptosis assay and found that 40 nM YM155 treatment for 24 h initiated apoptosis in LX2 cells (Supplementary Figure S2B). To confirm these results, we further evaluated the protein expression of caspase 3 and poly (ADP‐ribose) polymerase (PARP) by immunoblotting. 40 nM YM155 treatment induced caspase 3 activation and PARP cleavage in LX2 cells. Interestingly, a 10 nM dose of YM155 treatment for 24 h showed 30% cellular toxicity in LX2 cells and efficiently inhibited survivin protein expression without activating caspase 3 (Supplementary Figure S2C). Thus, we performed further experiments using 10 nM YM155 to investigate fibrogenic deactivation.

**FIGURE 2:**
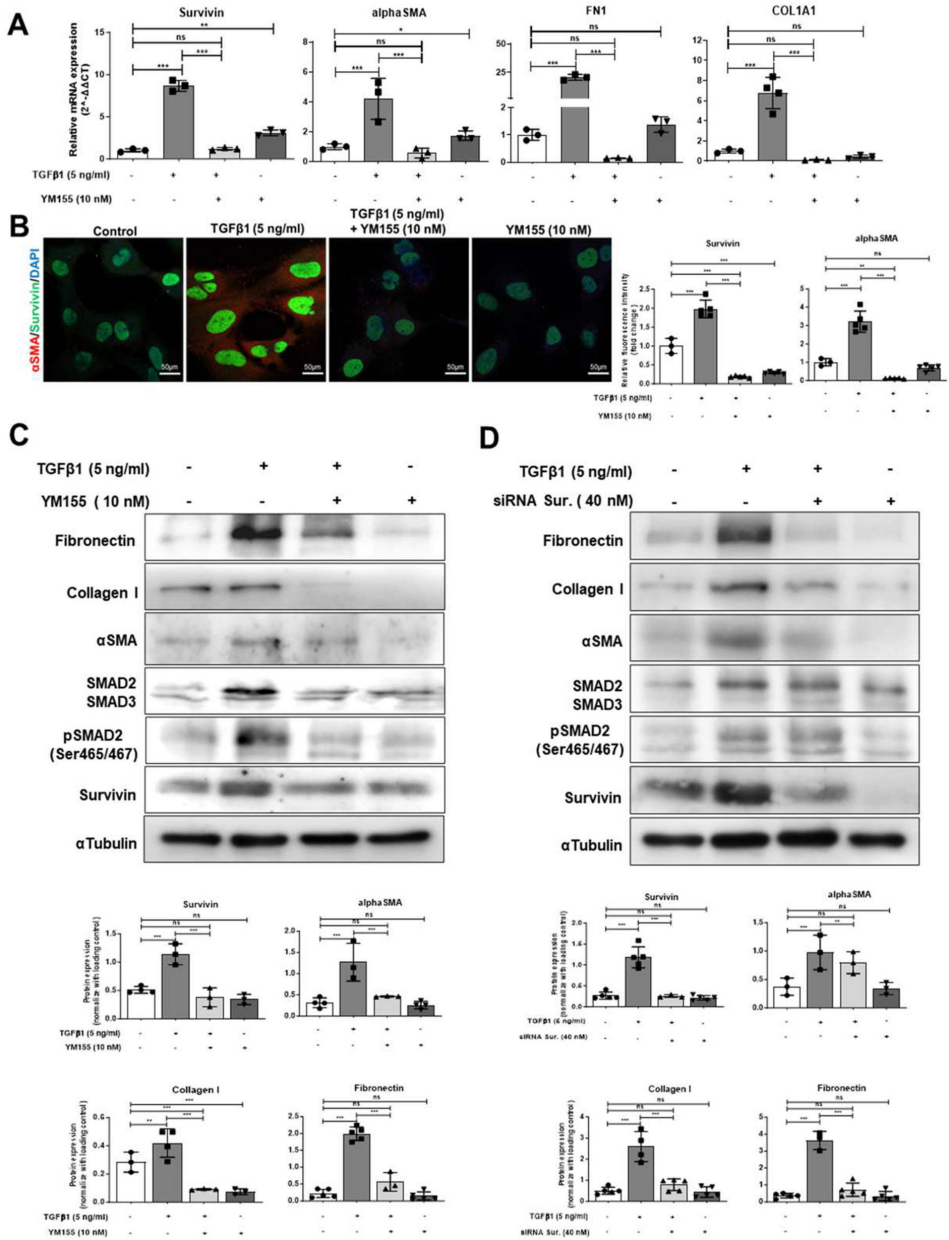
Survivin inhibition decreases the fibrogenic potential of activated HSCs. (A) Relative fold change in mRNA levels of survivin, *α*‐SMA, fibronectin, and collagen1 in TGF‐β1 (5 ng/mL) stimulated or control and YM155 (10 nM) treated or non‐treated LX2 cells after 24 h (B) IF image of TGF‐β1 (5 ng/mL) stimulated and TGF‐β1 (5 ng/mL) or control, and YM155 (10 nM) treated or non‐treated LX2 cells after 24 h representing survivin (green), *α*‐SMA (red), and nucleus (blue) staining (scale bar: 50 μm; 63x magnification). (C) Western blot after 24 h of TGF‐β1 (5 ng/mL) stimulated or control and YM155 (10 nM) treated or non‐treated LX2 cells. (D) Western blot after 24 h of TGF‐β1 (5 ng/mL) stimulated or control and siRNA survivin‐treated (40 nM) LX2 cells. Protein expression was quantified by analysis of band intensity normalized to α‐tubulin expression. nsP > 0.05, *P < 0.05 **P < 0.01, ***P < 0.001 [one‐way ANOVA for (A, B, C, D)].

To evaluate the effect of survivin inhibition on the fibrogenic activity of HSCs, we treated TGF‐β1 (5 ng/mL) stimulated LX2 cells with 10 nM YM155 for 24 h. Treated cells showed cell viability similar to non‐treated, unstimulated HSCs (Supplementary Figure S2D). However, TGF‐β1 stimulation increased the mRNA expression of survivin, *α*‐SMA, COL1A1, and fibronectin (FN1) in LX2 cells. Treatment of TGF‐β1 stimulated LX2 cells with 10 nM YM155 suppressed the mRNA expression of survivin, *α*‐SMA, COL1A1, and FN1 genes (Figure 2A). IF results confirmed that 10 nM YM155 treatment decreased the expression of both survivin and *α*‐SMA in TGF‐β1 stimulated LX2 cells (Figure 2B). Western blot analysis showed that TGF‐β1 (5 ng/mL) stimulation increased survivin, *α*‐SMA, collagen I, and fibronectin protein expression in LX2 cells. 10 nM YM155 treatment decreased survivin, αSMA, collagen I, and fibronectin protein levels in TGF‐β1‐stimulated LX2 cells. YM155 treatment also decreased the expression of SMAD2/3 and phosphorylated SMAD2 proteins, suggesting the involvement of TGF‐β1 signaling in the induction of survivin expression in HSCs (Figure 2C). We also silenced survivin expression in TGF‐β1 stimulated LX2 cells using specific siRNA. Annexin‐PI analysis of LX2 cells with siRNA‐mediated survivin knockdown showed no significant change in HSCs' viability (Supplementary Figure S3). Although siRNA mediated survivin inactivation in LX2 cells decreased the expression of *α*‐SMA, collagen I, and fibronectin in comparison to TGF‐β1 stimulated LX2 cells, it did not affect the SMAD2/3 and phosphorylated SMAD2 protein expression (Figure 2D), suggesting that survivin protein expression is mediated by TGFβ signaling.

### 2.3 Survivin inhibition limits the functional activity of activated HSCs

**FIGURE 3:**
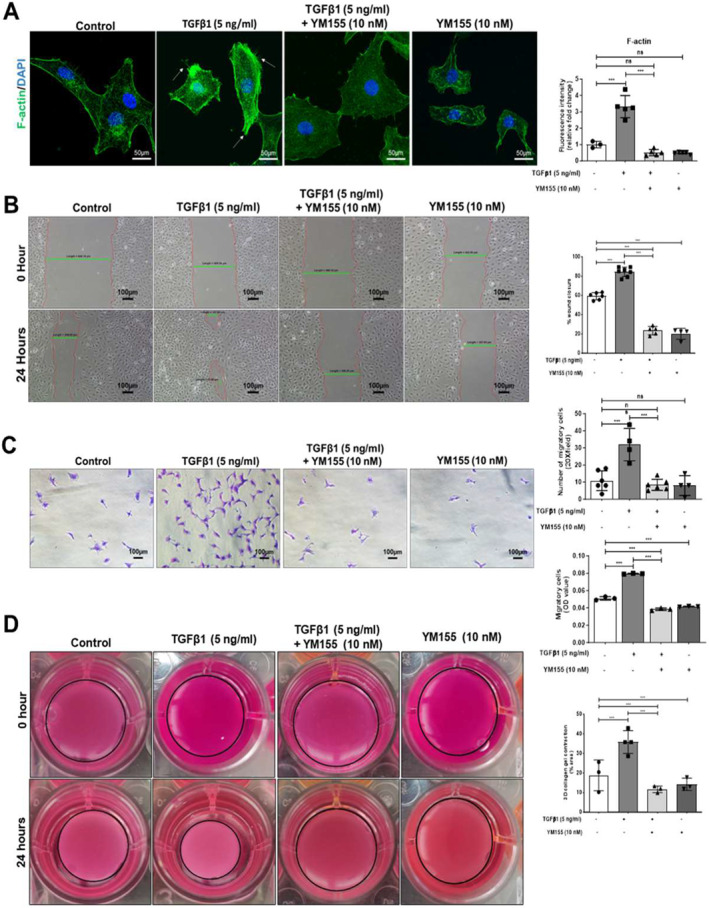
Survivin inhibition decreases the functional activity of activated HSCs. (A) IF images of F‐actin filament (green) rearrangement in LX2 cells stimulated with TGF‐β1 (5 ng/mL), followed by YM155 (10 nM) treatment for 24 h using Phalloidin and nucleus stain DAPI (blue) (scale bar: 50 μm; 63x magnification). (B) Wound healing cell migration (scratch) assay of TGF‐β1 (5 ng/mL) stimulated, TGF‐β1 (5 ng/mL) stimulated and YM155 (10 nM) treated LX2 cells after 24 h (scale bar: 100 μm; 10x magnification). (C) Trans‐well cell migration assay of TGF‐β1 (5 ng/mL) stimulated and YM155 (10 nM) treated LX2 cells after 24 h (scale bar: 500 μm; 20x magnification). (D) 3D gel collagen contraction assay representing collagen contraction ability of embedded LX2 cells treated with TGF‐β1 (5 ng/mL) or TGF‐β1 (5 ng/mL) with YM155 treatment for 24 h nsP > 0.05, *P < 0.05, **P < 0.01, ***P < 0.001 [one‐way ANOVA for (A, B, C, D)].

Cytoskeleton filament rearrangement in HSCs regulates their contraction ability, which affects their migration.^20^, ^21^ To investigate the effect of survivin inhibition on cytoskeleton rearrangement, we stained F‐actin filaments using phalloidin fluorescence dye and performed IF studies. TGF‐β1 (5 ng/mL) stimulation altered the cytoskeleton rearrangement forming stress fibers in LX2 cells. YM155 treatment in TGF‐β1 stimulated cells reduced the F‐actin expression and their rearrangement in association with a reduction in stress fiber formation (Figure 3A). To examine the migration ability of HSCs, we performed a scratch migration assay (wound closure assay). As expected, TGF‐β1 (5 ng/mL) stimulation enhanced the migration of LX2 cells compared to unstimulated cells. However, YM155 treatment limited the migration ability of TGF‐β1 stimulated LX2 cells (Figure 3B). To confirm these results, we also performed a trans‐well migration assay and found that 10 nM YM155 treatment reduced migration in TGF‐β1 stimulated LX2 cells across a porous membrane in trans‐well chambers (Figure 3C).

Next, we measured the effect of survivin inhibition on HSC contraction ability through a 3D collagen gel contraction assay. TGF‐β1 (5 ng/mL) stimulation enhanced the contraction ability of LX2 cells as shown by the shrunken 3D collagen gel. However, YM155 treatment of the TGF‐β1 stimulated cells reduced the shrinkage of 3D collagen gel indicating their decreased contraction ability (Figure 3D). These results suggest that survivin inhibition decreases the migration and contraction properties of activated HSCs while also suppressing their fibrogenic activation.

### 2.4 Survivin inhibition induces senescence and cell cycle arrest at the G2/M phase in activated HSCs

**FIGURE 4:**
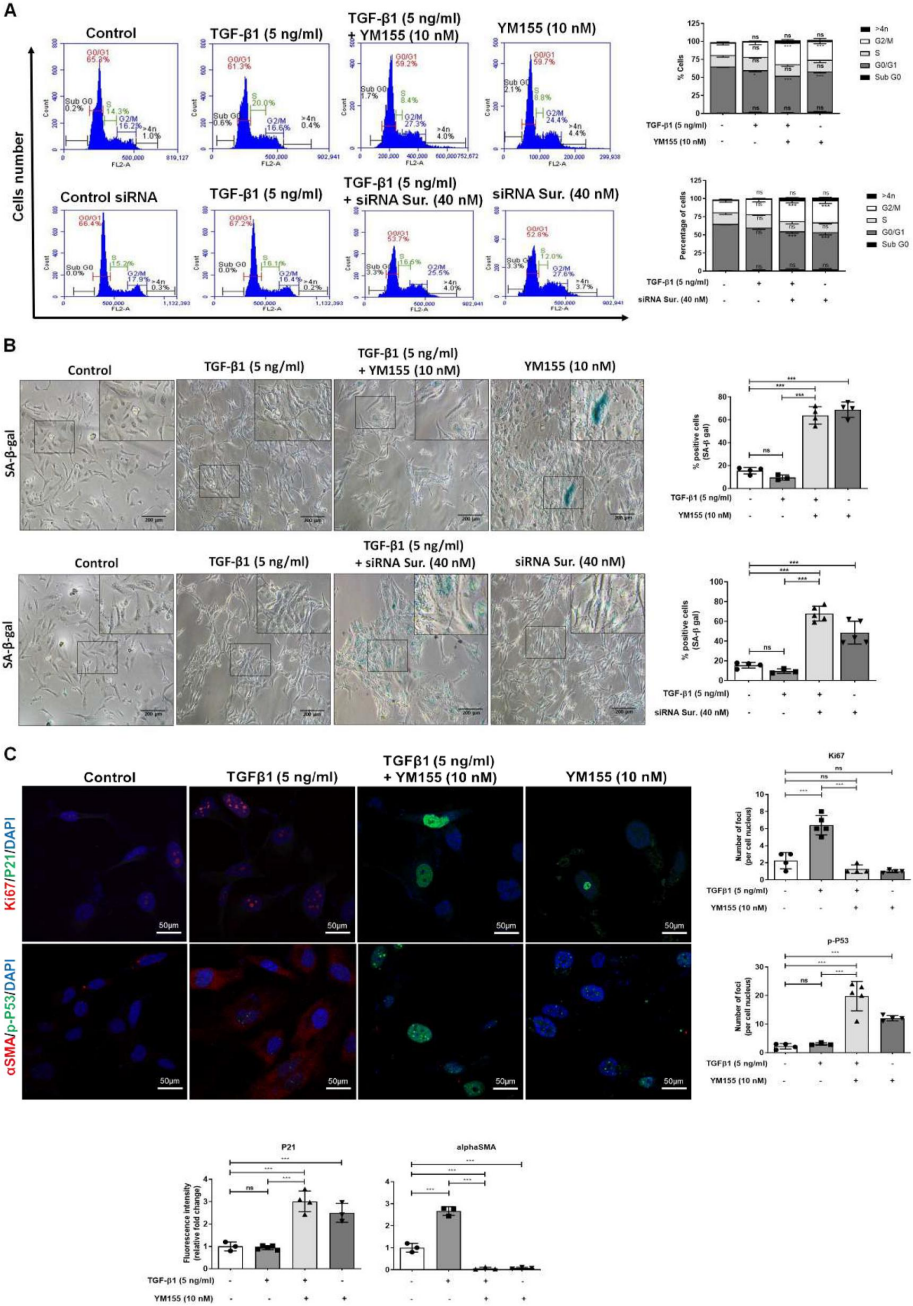
Survivin inhibition induces senescence and cell cycle arrest at G2/M phase in activated HSCs. (A) Flow cytometry‐based cell cycle analysis using propidium iodide (PI) in LX2 cell stimulated with TGF‐β1 (5 ng/mL) alone or TGF‐β1 (5 ng/mL) stimulated LX2 cells followed by YM155 (10 nM) or siRNA survivin (40 nM) treatment. (B) *β*‐galactosidase (β‐SA‐gal) senescence assay performed with TGF‐β1 stimulated or TGF‐β1 (5 ng/mL) stimulated and YM155 (10 nM) or siRNA survivin (40 nM) treated LX2 cells (scale bar: 100 μm; 10x magnification). (C) IF images of senescent LX2 cells representing Ki67 (red), P21 (green), *α*‐SMA (red), p‐P53 (green), and nucleus (blue) staining in TGF‐β1 stimulated or TGF‐β1 (5 ng/mL) and YM155 (10 nM) treated LX2 cells (scale bar: 50 μm, 63x magnification). [one‐way ANOVA for (B, C); two‐way ANOVA for (A)].

Survivin protein regulates the cell cycle and a defect in its expression results in cell cycle arrest.^22^ To examine whether survivin suppression can induce a cell cycle arrest in activated HSCs, we inactivated survivin through YM155 treatment or siRNA‐mediated knockdown in LX2 cells and cultured them for 5 days in YM155 or siRNA‐free media. PI‐flow cytometry‐based cell cycle analysis revealed that survivin suppression induced cell cycle arrest at the G2/M phase in TGF‐β1 stimulated LX2 cells compared to unstimulated control (Figure 4A). The arrested cells became enlarged and multinucleated, suggesting that survivin inhibition induces G2/M phase cell cycle arrest and polyploidy in activated HSCs.

Usually, senescent cells display an enlarged, multinucleated phenotype and play an important role in the fibrogenic deactivation of HSCs, which eventually promotes the resolution of liver fibrosis.^16^, ^23^ To analyze senescence in survivin inactivated LX2 cells, we performed X‐gal based *β*‐galactosidase (SA‐β‐gal) assay. SA‐β‐gal enzyme expression is a hallmark of senescence that yields blue precipitation of X‐gal (5‐Bromo‐4‐Chloro‐3‐Indolyl *β*‐D‐Galactopyranoside).^16^ Survivin suppression through YM155 treatment or siRNA‐mediated knockdown induced senescence in TGF‐β1 stimulated LX2 cells and showed more blue precipitation than nonstimulated control (Figure 4B). Fluorescence probe flow cytometry‐based SA‐β‐gal assay further confirmed that survivin suppression increased the percentage of senescent cells in the total population (Supplementary Figure S4A). These results indicate that survivin inhibition induces cell cycle arrest and senescence in activated HSCs.

We further analyzed the expression of molecules related to cell cycle arrest such as P21, phosphorylated P53 (p‐P53), and the proliferative marker, Ki67, in survivin inactivated LX2 cells. The senescent cells exhibited high expression of P21 and p‐P53 and low expression of Ki67.^16^ IF images showed that survivin suppression through YM155 treatment or siRNA silencing in TGF‐β1 stimulated LX2 cells induced a higher P21 and lower Ki67 nuclear expression than non‐treated cells (Figure 4D,E, and Supplementary Figure S4B). Phosphorylated P53 acts as a transcription factor and is present upstream of P21. Survivin inactivated senescent cells showed more p‐P53 punctae in their nuclei and lower expression of *α*‐SMA compared to non‐treated cells (Figure 4D,E, and Supplementary Figure S4B). These results suggest that targeting survivin in activated HSCs induces cell cycle arrest and senescence that collectively results in fibrogenic deactivation.

### 2.5 M2 macrophages increase survivin expression through the TGF‐β‐TGF‐β receptor‐I/II axis in HSCs

**FIGURE 5:**
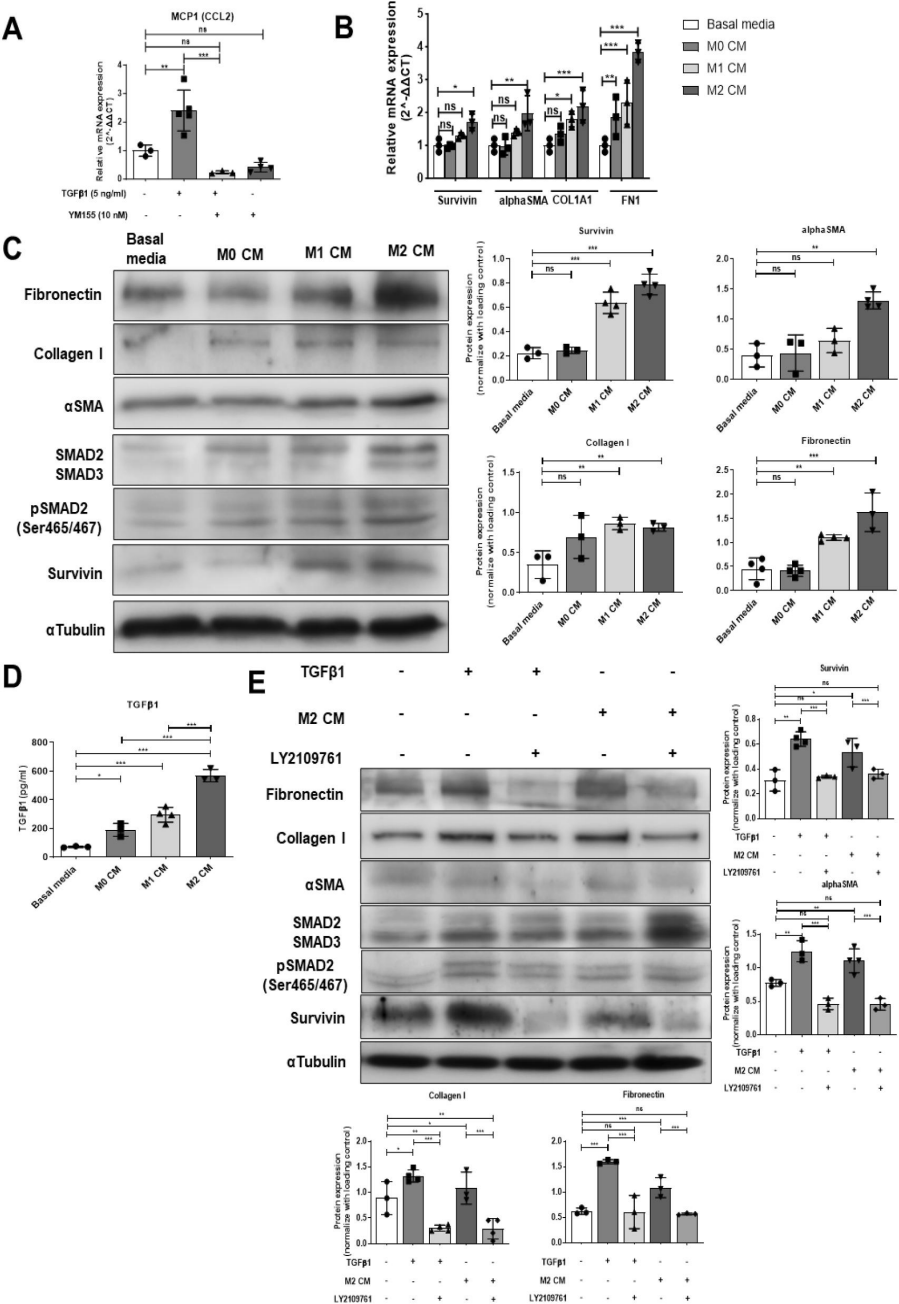
M2 macrophage increases survivin expression and fibrogenic activity in HSCs. (A) Relative mRNA expression of MCP1 (CCL2) gene in TGF‐β1 (5 ng/mL) stimulated or TGF‐β1 (5 ng/mL) with YM155 (10 nM) treated LX2 cells after 24 h analyzed by qPCR. (B) Relative mRNA expression of LX2 cells cultured in the presence of media conditioned by polarized macrophages for 24 h analyzed by qPCR. (C) Western blot representing protein expression in LX2 cells cultured in conditioned media (CM) of polarized macrophages for 24 h. (D) Secreted TGF‐β1 cytokine levels in CM of polarized macrophages at 24 h analyzed by ELISA. (E) Western blot representing protein expression in LX2 cells treated with M2 CM and TGF‐β1 RI/II receptor inhibitor, LY2109761 (10 μM) for 24 h, TGF‐β1 (5 ng/mL) alone, and TGF‐β1 (5 ng/mL) with LY2109761 (10 μM) treated LX2 cells for 24 h. nsP > 0.05, *P < 0.05, **P < 0.01, ***P < 0.001 [one‐way ANOVA for (A, C, D, E); two‐way ANOVA for (B)].

Activated HSCs secrete the monocyte chemoattractant protein‐1 (MCP1/CCL2) responsible for the infiltration of macrophages from the bone marrow, and plays a vital role in liver fibrosis progression.^24^, ^25^ We found that TGF‐β1 stimulation increased the mRNA expression of CCL2 in LX2 cells and YM155‐mediated survivin inhibition decreased the expression (Figure 5A). To investigate the role of polarized macrophages in regulating survivin expression in HSCs, we first differentiated the human monocyte THP1 cell line into macrophages (M0 macrophage), followed by polarizing them into M1 and M2 macrophages. The phenotype of polarized macrophages was confirmed by analyzing the mRNA expression of different surface makers and cytokines through qPCR. M0 macrophages showed higher CD14 and CD68 mRNA expression than non‐differentiated monocytes. However, M1‐polarized macrophages exhibited high mRNA expression of CD80, CD86, and pro‐inflammatory cytokines such as TNF‐α, IL‐1β, and IL‐6. On the other hand, M2‐polarized macrophages exhibited high mRNA expression of CD206, CD209, and anti‐inflammatory cytokines, IL‐10, FN1, and TGF‐β1 (Supplementary Figure S5A and S5B). Next, we cultured LX2 cells in polarized macrophage conditioned media (CM) for 24 h. M1 and M2 macrophage CM alone was sufficient to increase the mRNA expression of FN1 and COL1A1 in HSCs; however, only M2 macrophage CM significantly increased the *α*‐SMA and survivin expression in LX2 cells (*p* < 0.05) (Figure 5B). Immunoblot analysis further confirmed that M2 macrophage CM induced survivin protein expression in LX2 cells and also enhanced *α*‐SMA, collagen I, and fibronectin protein expression. Interestingly, M2 macrophage CM also upregulated SMAD2/3, and phosphorylated SMAD2 in LX2 cells, indicating the possible involvement of M2 macrophage‐derived TGF‐β1 in survivin induction (Figure 5C). To confirm, we quantified the TGF‐β1 cytokine levels secreted by macrophages in their CM through ELISA. M2‐polarized macrophage CM showed maximum TGF‐β1 cytokine levels in comparison to M0 and M1 macrophages (Figure 5D).

To analyze whether M2 macrophage‐derived TGF‐β1 stimulates survivin expression in HSCs, we pre‐treated LX2 cells with TGF‐β receptor‐I/II inhibitor, LY2109761 followed by culturing them in M2 macrophage CM for 24 h. M2 macrophage CM induced survivin expression in LX2 cells decreased with the pre‐treatment of LY2109761 (Figure 5E). These results suggest that M2‐polarized macrophages induce survivin expression and fibrogenic activation in HSCs via TGF‐β1 cytokines.

### 2.6 YM155‐mediated survivin suppression ameliorates liver fibrosis and induces HSC senescence in vivo

To validate the in vitro findings, we developed a progressive liver fibrosis model in male BALB/c mice by repeated intraperitoneal *(i*.*p)* injections of CCl_4_ (1.0 mL/kg mice body weight) for 6 weeks. The treatment group was given five doses of YM155 (10 mg/kg mice body weight) by *i*.*p* injections from the 5th–6th week of CCl_4_ injection and the mice were sacrificed within 24 h of the last dose. Hepatotoxic markers, ALT (Alanine Aminotransferase), and AST (Aspartate Aminotransferase) in blood serum confirmed the injury in CCl_4_ administered mice liver. YM155 treatment showed decreased ALT and AST levels indicating an improvement in hepatoxicity (Figure 6A and Supplementary Figure S6). Hematoxylin and eosin (H&E) staining showed prominent hepatocyte degeneration, necroinflammation, and infiltration of mononuclear immune cells in the periportal region of the injured liver. Sirius red staining represented more collagen deposition in the injured liver of the CCl_4_ group compared to healthy controls. However, YM155 treatment improved the histopathological parameters and showed less deposition of collagen. Staining of collagen I by IHC further confirmed the low collagen deposition in the periportal region of YM155 treated compared to non‐treated fibrotic mice liver (Figure 6B).

FIGURE 6Survivin inhibition induces cellular senescence in HSCs and ameliorates liver fibrosis in vivo. (A) A schematic diagram of protocol for developing liver fibrosis model and YM155 treatment in mice. Liver injury biomarkers, AST, and ALT measured in mice blood serum. (B) Photographic images, Sirius red staining, H&E staining, and IHC of mice livers (scale bar: 50 μm, 4x magnification). (C) Relative hepatic mRNA expression of specific genes in control (olive oil), CCl_4_, and CCl_4_ with YM155‐treated mice livers. (D) Western blot representing protein expression in control (olive oil), CCl_4_, and CCl_4_ with YM155‐treated mice livers. (E) IHC and IF images representing survivin (green), αSMA (red), and nucleus (blue) in control (olive oil), CCl_4,_ and CCL_4_ with YM155‐treated mice livers (scale bar: 50 μm, 20x, and 40x magnification). (F) *β*‐SA‐gal senescence assay in control (olive oil), CCl_4_, and CCl_4_ with YM155‐treated mice livers. IHC representing P21, Ki67, and phosphorylated P53 staining in mice liver sections (scale bar: 50 μm, 20x magnification). All mice experiments were carried out with minimum *n* = 3 mice with two replicates each. nsP > 0.05, *P < 0.05, **P < 0.01, ***P < 0.001 [one‐way ANOVA for (A, B, C, D, E, F); two‐way ANOVA for (D)].

**Figure:**
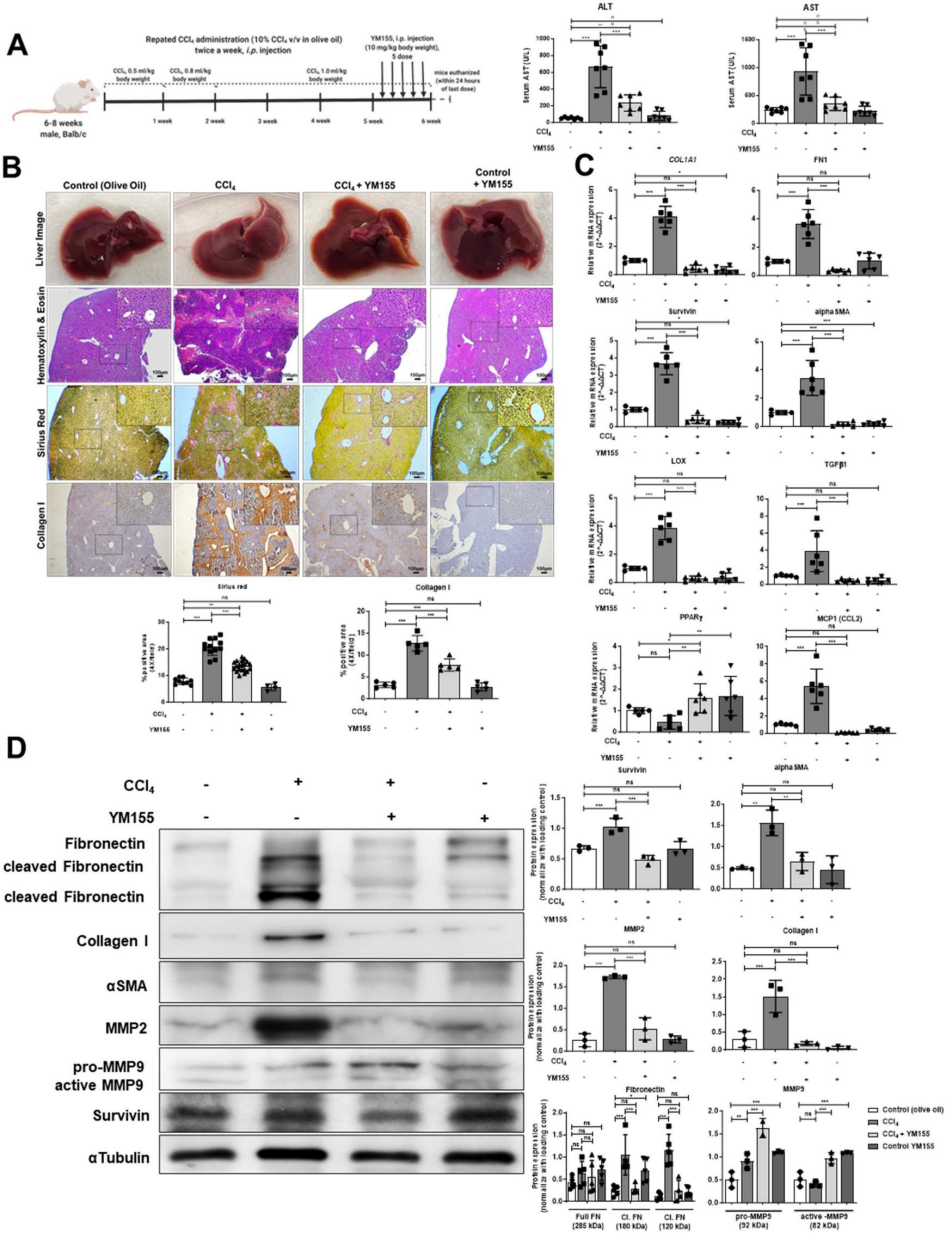

**Figure:**
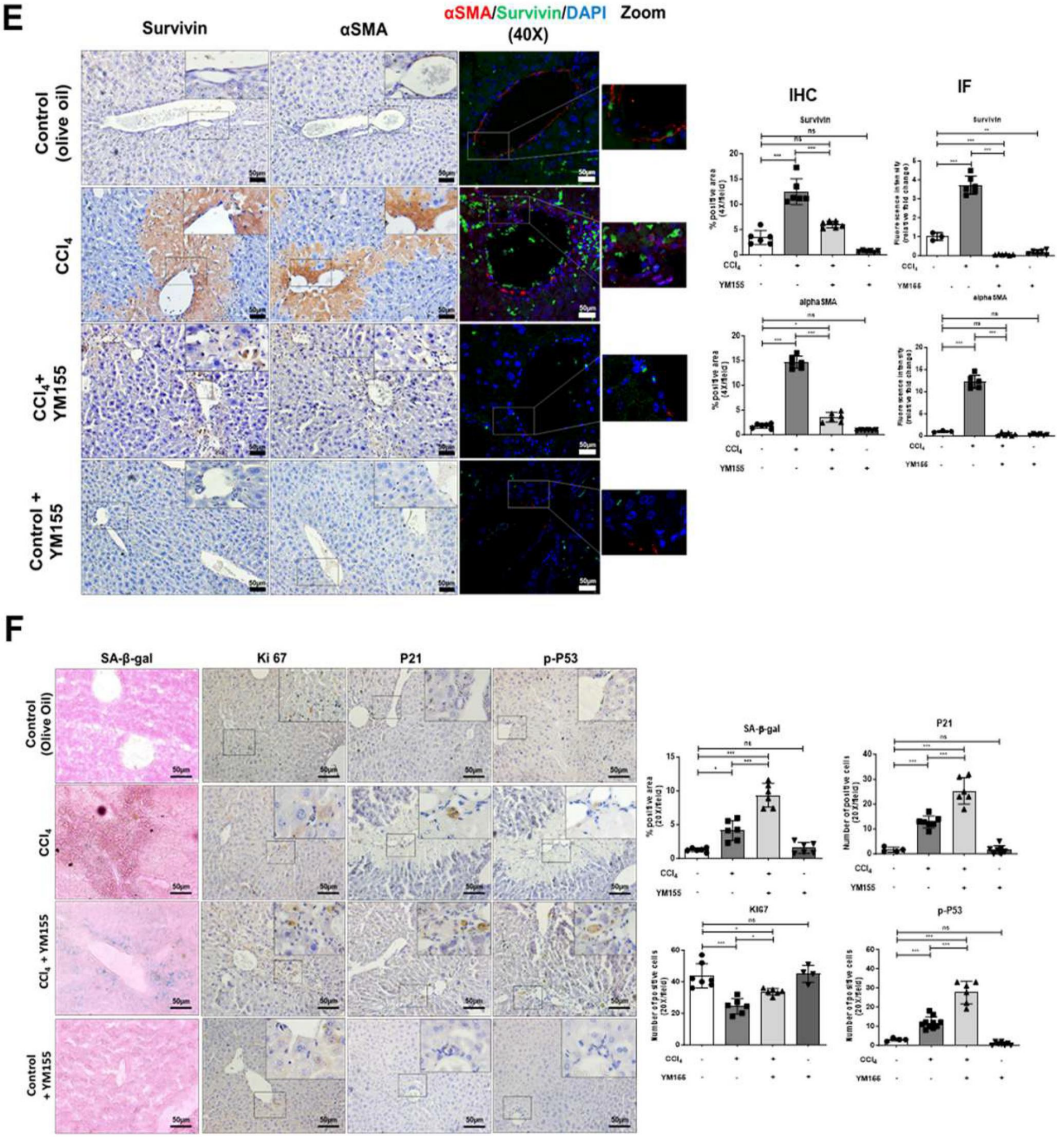

Next, we analyzed the hepatic expression of survivin and other fibrogenesis‐related genes. Survivin mRNA expression along with *α*‐SMA, COL1A1, FN1, LOX, and TGF‐β1 was found to increase in the fibrotic liver. However, the expression of these genes decreased with the treatment of YM155. PPAR‐γ gene expression, a marker of deactivated HSCs, was found low in the fibrotic liver.^26^ Conversely, PPAR‐γ expression increased with the YM155 treatment, suggesting the possibility that survivin inhibition in the fibrotic liver reverses the activation of HSCs. Interestingly, macrophage infiltrating CCL2 chemokine expression was found suppressed in YM155 treated fibrotic liver (Figure 6C). We further evaluated the expression of hepatic proteins and found increased survivin, *α*‐SMA, collagen I, and fibronectin protein expression in the fibrotic liver, whereas YM155 treatment decreased their expression. In the fibrotic liver, fibronectin protein is predominately found in a cleaved form. MMP2 is reported to be responsible for the cleavage of fibronectin into fragments and for the progression of liver fibrosis.^27^ Conversely, MMP9 promotes HSC apoptosis and promotes the resolution of the fibrotic liver.^28^ Concurring with these reports, MMP2 protein expression was increased and MMP9 was decreased in the fibrotic liver. However, survivin suppression decreased MMP2 expression and increased MMP9 expression in YM155‐treated mice fibrotic liver (Figure 6D). These results suggest that survivin suppression decreased HSC activation and other fibrogenesis‐related genes in the fibrotic liver.

We also analyzed the localization of *α*‐SMA and survivin in the injured liver through IHC. Healthy mice liver showed minimal survivin and *α*‐SMA expression; however, in the injured liver, their expression was found significantly increased in the periportal region. YM155 treatment decreased both survivin and *α*‐SMA expression at the injury site, whereas in the hepatocytes survivin expression was found negligible. IF staining confirmed that survivin expression was confined to *α*‐SMA^+^ cells, and YM155 treatment decreased their expression (Figure 6E). These results suggest that survivin expression increases in activated HSCs in the fibrotic liver, and its suppression limits their activation, which ultimately reduces liver fibrosis progression.

Next, we investigated whether survivin inhibition also induces senescence in fibrotic mice livers by performing an SA‐β‐gal assay. YM155‐mediated survivin suppression increased the number of senescent cells at the injury site compared to non‐treated fibrotic mice liver. We also analyzed the expression of p‐P53 and P21, cell cycle arrest markers in YM155‐treated fibrotic mice liver, and found that survivin suppression increased their expression in the periportal region. However, Ki67 expression was minimally elevated in YM155‐treated fibrotic mice liver (Figure 6F). Thus, survivin suppression increased senescence in activated HSCs that ameliorates fibrosis in chronic liver injury.

### 2.7 Survivin suppression decreases pro‐inflammatory gene expression and depletes macrophage population in fibrotic liver

**FIGURE 7:**
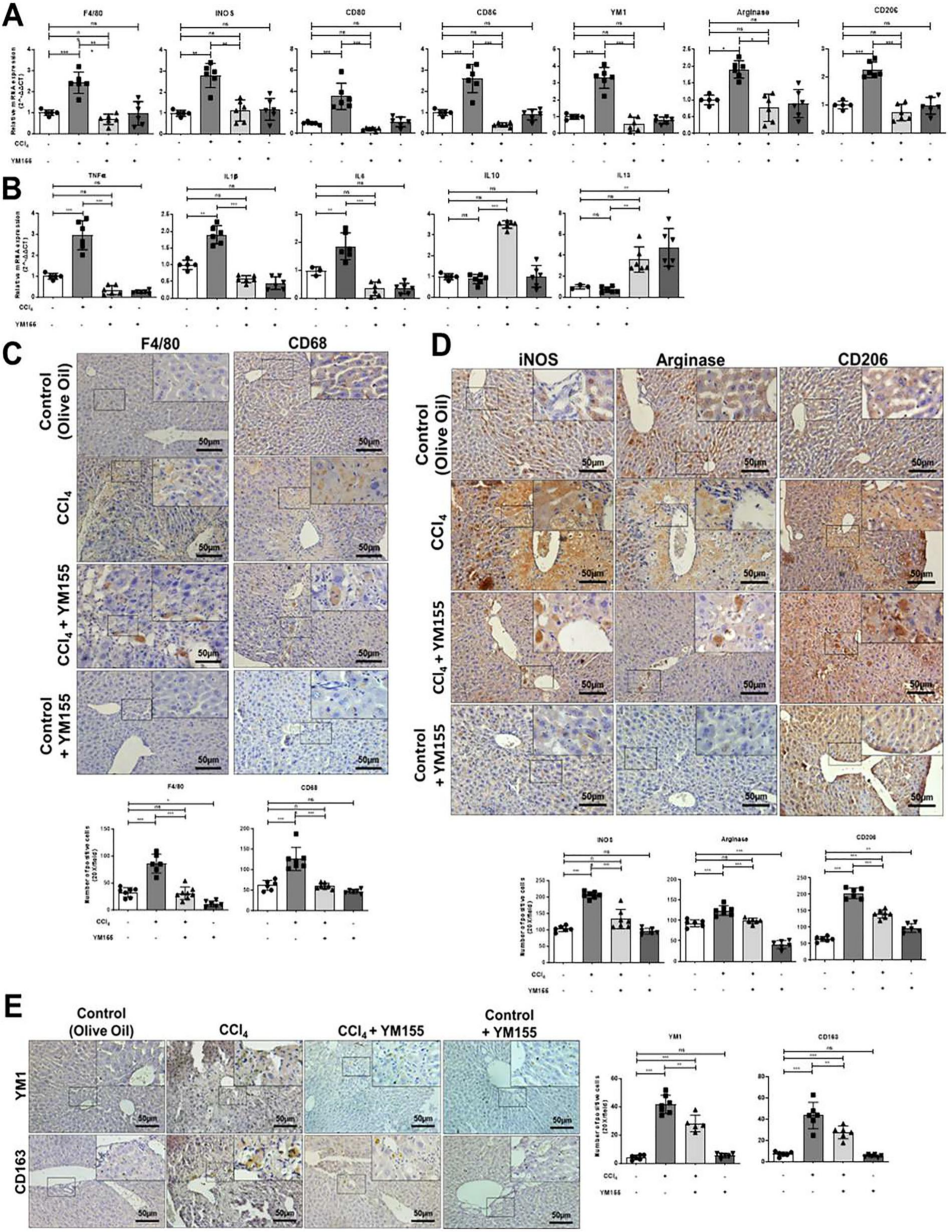
Survivin inhibition decreases monocyte and macrophage localization in the fibrotic liver. (A) Relative hepatic mRNA expression of F4/80, a marker for macrophages; CD80, CD86, and iNOS, markers of M1‐polarized macrophages; YM1, arginase, CD206, makers of M2‐polarized macrophages in control (olive oil), CCl_4_, and CCl_4_ with YM155‐treated mice livers. (B) Relative hepatic mRNA expression of pro‐inflammatory, TNF‐α, IL‐1β, IL‐6 cytokines, and anti‐inflammatory, IL‐10, IL‐13 cytokines. (C) IHC of mice liver sections representing F4/80 and CD68 staining. (D) IHC representing iNOS, arginase, and CD206 staining and, (E) IHC showing YM1 and CD163 staining. (Scale bar: 50 μm, 20x magnification) All mice experiments were carried out with minimum *n* = 3 mice with two replicates each. ns*P* > 0.05, *P < 0.05, **P < 0.01, ***P < 0.001 [one‐way ANOVA for (A, B, C, D, E)].

We evaluated the effect of YM155 on hepatic macrophages in mice fibrotic livers. mRNA expression of macrophage marker, F4/80, was found to be upregulated in the fibrotic liver. In addition, M1‐polarized macrophage markers, CD80, CD86, and iNOS as well as M2‐polarized macrophage markers, YM1, arginase, and CD206 were also found overexpressed. YM155 treatment suppressed the expression of markers associated with both M1‐ and M2‐polarized macrophages (Figure 7A). The fibrotic liver showed high expression of pro‐inflammatory cytokines such as TNF‐α, IL‐1β, IL‐6, and TGF‐β1 and low expression of anti‐inflammatory cytokines such as IL‐10 and IL‐13. YM155 treatment decreased the mRNA expression of pro‐inflammatory cytokines and increased the anti‐inflammatory cytokines in the treated livers (Figure 7B).

We also performed IHC to locate the macrophages in the liver tissues and found a high number of F4/80^+^ and CD68^+^ cells at the injury site of the fibrotic liver. However, YM155 treatment decreased the F4/80^+^ and CD68^+^ cells in the portal region, and they were found throughout the fibrotic septa (Figure 7C). Based on the expression of characteristic markers, we observed both M1 (iNOS) and M2 (arginase, CD206) polarized macrophages at the injury site in the fibrotic livers. Interestingly, survivin suppression in the fibrotic liver decreased iNOS, arginase as well as CD206 expression. However, survivin inhibition reduced iNOS expression by a comparatively higher proportion than that of arginase and CD206 (Figure 7D). We also analyzed YM1 and CD163 expression, characteristic of mouse M2 macrophages. YM1 and CD163 expression were found upregulated in the fibrotic mice liver and YM155 treatment decreased their expression (Figure 7E). These results suggest that survivin suppression depletes macrophage population, which promotes resolution of fibrosis in injured liver.

## 3 DISCUSSION

In this study, we aimed to limit liver fibrosis progression in chronic injury and modulate its fibrotic microenvironment by deactivating fibrogenic HSCs. In our investigation, we found that an increase in survivin expression was associated with the activation of HSCs. Survivin expression was also found upregulated with fibrosis progression in human and mice liver tissues following injury. Fundamentally, survivin protein participates in the cytokinesis of proliferating cells, promoting hepatocyte growth and liver regeneration.^12^ Previous studies have interestingly revealed that suppression of survivin in hepatocytes did not alter the liver architecture and function but rather enhanced hepatocyte polyploidy.^14^ Studies have highlighted the beneficial advantage of hepatocyte polyploidy in protecting the liver from hepatic damage and tumorigenesis.^29^ However, the role of survivin in chronically injured liver and fibrosis is largely unknown. Our recent study reported that survivin overexpression is associated with HSC activation, following the initiation of liver injury. Survivin protein is involved in cell survival and proliferation of early‐activated HSCs and fibrosis perpetuation. Survivin suppression in the early phase of HSC activation limits their fibrogenic response and delays the onset of fibrosis in the injured liver.^5^ Based on these findings, in the present study, we investigated the therapeutic potential of targeting survivin in activated HSCs in a fully developed fibrotic liver. We targeted survivin in activated HSCs or myofibroblasts, limiting their fibrogenic response in fully developed fibrosis underlying chronic liver injury without exacerbating the hepatocellular injury. We found that survivin expression is localized to αSMA^+^ HSCs in the fibrotic area with minimal hepatocyte expression in chronically injured human and mice livers.

In the present study, we reported that targeting survivin in activated HSCs inhibits its ECM protein expression, proliferation, migration, and contraction ability, the key features of fibrogenic HSCs, which eventually halt liver fibrosis progression. Survivin suppression through YM155 decreased the population of αSMA^+^ HSCs and collagen I expression, which improved liver injury compared to non‐treated fibrotic mice. We found that survivin inhibition induces cell cycle arrest at the G2/M phase in HSCs and enhanced polyploidy. Survivin inactivated cells exhibited high expression of cell cycle arrest markers, P21 and p‐P53, and low expression of a proliferative marker, Ki67, which eventually induced senescence. Previous studies have reported that senescence in activated HSCs plays a vital role in their fibrogenic deactivation and liver fibrosis resolution.^16^ In mice fibrotic liver, survivin suppression induces senescence in periportal HSCs that restricts fibrosis progression following liver injury. Studies have demonstrated additional cellular pathways by which survivin regulates HSC phenotype and function, and plays a role in liver fibrosis. A study revealed that high fructose diet promotes zinc finger E‐box binding homeobox 1 (ZEB1) nuclear translocation that decreases microRNA‐203 (miR‐203) expression in rat livers. miR‐203 low‐expression increases survivin expression that further activates TGF‐β1/Smad signaling and liver fibrosis.^30^ Another study showed marked activation and proliferation of hepatic stellate cells in liver recovery after ischemia‐reperfusion injury in murine model. During liver repair, YAP (yes‐associated protein 1) and TAZ (transcriptional coactivator with PDZ‐binding motif) and their target gene, survivin were activated selectively in hepatic stellate cells. Treatment of mice with verteporfin, an inhibitor of YAP and TAZ, decreased hepatic stellate cell proliferation, survivin, and cardiac ankyrin repeat protein expression in association with a significant decrease in hepatocyte proliferation.^31^

Many immune cell populations are involved in the pathogenesis of fibrosis with diverse functions. The interactions between immune cells and myofibroblasts are key events in the fibrogenic responses. Activated immune cells produce multiple cytokines that modulate the differentiation, proliferation, survival, and collagen production of myofibroblasts. Moreover, most immune cell types are heterogeneous with functional plasticity modulated by both systemic and microenvironmental factors. Thus, the cellular identities and local niches are of key significance for their functions in fibrosis. Hepatic macrophages are key immune cells implicated in promoting liver fibrosis as well as fibrosis resolution. The initiation and persistence of HSC activation is directly regulated by hepatic macrophages. Macrophages activate HSCs and promote the progression of liver fibrosis.^32^ On the other hand, during the reversal of liver fibrosis, macrophages can drive HSC apoptosis and ECM degradation.^33^ In the injured liver, dying hepatocytes activate Kupffer cells (KC) and HSCs to secrete MCP1/CCL2 chemokines responsible for the infiltration of macrophages from the bone marrow.^34^ Depending on the context, the fibrotic microenvironment drives the differentiation of infiltrated macrophages to pro‐inflammatory M1 polarized or anti‐inflammatory M2‐polarized macrophages.^35^ It has been reported that HSCs promote hepatic macrophage infiltration through the CCL2/CCR2 pathway and induce M2 polarization to aggravate liver fibrosis.^36^ In this study, we found that M2‐polarized macrophages activate the TGF‐β receptor I/II in HSCs via secreted TGF‐β1 cytokine, which enhanced survivin expression and activation of HSCs. Survivin suppression desensitizes the HSCs toward TGF‐β1‐mediated activation. Surprisingly, it also affected the macrophage population in the liver by regulating CCL2 expression that alters the hepatic inflammatory microenvironment and protects from liver injury.^24^ YM155 treatment decreases both M1 and M2‐polarized macrophage accumulation at the injury site of the fibrotic liver. However, it is important to highlight that the M1‐polarized macrophage population was reduced to a higher extent than M2‐polarized macrophages. These findings are in concordance with a previous study that demonstrated that both M1 and M2 macrophage population was significantly increased during liver fibrosis; however, during fibrosis regression, M1 macrophages may play a more important role in the regression of liver fibrosis than M2 macrophages.^37^ The reduction of macrophage subtypes in the fibrotic liver also affected the cytokine expression in liver tissues showing a decrease in pro‐inflammatory cytokines compared to anti‐inflammatory cytokines such as IL‐10 and IL‐13. Recent studies have highlighted that CD206^+^ M2‐polarized macrophage infusion protects the liver from acute injury and their phagocytic activity clears hepatic debris, promoting hepatocyte proliferation.^18^ In our study, CD206^+^ M2 macrophages were found abundantly at the resolved site of the fibrotic septa in YM155‐treated mice liver. These observations suggest that M2‐polarized macrophages were possibly involved in removing hepatic debris and collagen degradation through their phagocytic activity that promoted liver tissue repair. Further studies on the liver immune landscape will allow a better understanding of the implications of other key players of the innate and acquired immunity, during fibrosis development and resolution.

In conclusion, the present study demonstrates survivin as a pro‐fibrogenic protein that is upregulated during the fibrogenic activation of HSCs in the injured liver. Targeting of survivin in HSCs decreases their fibrogenic response by inducing cellular senescence. Survivin suppression also affects the population of macrophage subtypes, which collectively ameliorates liver fibrosis. Therefore, targeting survivin in activated HSCs provides a novel approach for the treatment of fibrosis in chronic liver injury.

## 4 METHODS

### 4.1 Human liver biopsy samples

Paraffin‐fixed human liver biopsy samples with pre‐determined fibrosis stage based on METAVIR score irrespective of etiology were obtained from the Biobank of the Institute of Liver and Biliary Sciences (ILBS), India. We categorized human liver biopsy samples into etiology independent F0/F1 METAVIR (*n* = 10; no fibrosis/mild fibrosis), F2/F3 METAVIR (*n* = 10; advance fibrosis), and F4 METAVIR (*n* = 8; cirrhosis). All procedures were carried out as per approval and guidelines (ILBS/IEC/IRB‐39/M5) by the Institute Ethics Committee and Institute of Liver and Biliary Sciences.

### 4.2 Primary mice HSC isolation

Primary mouse HSCs were isolated from healthy BALB/c (10 weeks age) male mice (*n* = 3) through in situ liver perfusion.^38^ Ultrapure population of qHSCs was collected through BD FACSAria III cell sorter for retinoid detection using 405–407 nm laser.

### 4.3 Animal model of advanced chronic liver disease

Animal care experimental procedures were carried out as per guidelines of Control and Supervision of Experiments on Animals (CPCSEA), Govt. of India. The study (CPCSEA/IAEC/2018/05/01) was approved by the Institutional Animal Ethics Committee (IAEC), Amity University Uttar Pradesh. Male BALB/c mice (6–8 weeks age‐old) were purchased from the National Institute of Biologicals (NIB), India, and segregated into four groups (each *n* = 7). To develop liver fibrosis, mice were administered with increasing concentration of carbon tetrachloride (CCl_4_) prepared in olive oil (week 1: 0.5 mL/kg; week 2: 0.8 mL/kg and week 3–6: 1 mL/kg of body weight) twice a week by intraperitoneal (*i*.*p*) injections for 6 weeks. Non‐fibrotic mice injected only with olive oil were used as controls. In the treatment groups, mice were treated with five doses of survivin inhibitor, YM155 (10 mg/kg of body weight) every alternate day, beginning from the 5^th^ week to the 6^th^ week of CCL_4_ injection. The mice were sacrificed at the 6^th^ week and liver tissues were collected and stored for subsequent analysis.

### 4.4 Statistical analysis

All data were presented as mean ± S.D. (standard deviation) from at least three separate independent experiments. Differences among groups were tested for statistical significance by Student's *t*‐test; when comparing 2 groups, by one‐way ANOVA; when comparing multiple conditions with repeated measures by two‐way ANOVA using Prism‐GraphPad. Differences were considered significant at *p* < 0.05. For all the tests, significant values were *P* < 0.05. Full details of other methods are mentioned in the supplementary information.

## AUTHOR CONTRIBUTIONS

Sachin Sharma designed the experiments, performed most of the biochemical and cellular experiments, acquired, analyzed, and interpreted the data with statistical validation. Shaikh Maryam Ghufran supported experimentation, especially animal work. Mehreen Aftab performed bioinformatics analyses with publicly available datasets. Chhagan Bihari provided human samples and helped with immunohistochemistry studies. Sampa Ghose carried out ELISA, qPCR experiments, and critical revision of the manuscript. Subhrajit Biswas was involved in the study concept and design, acquisition, analysis, and interpretation of data; drafting of the manuscript, and critical revision of the manuscript for intellectual content.

## CONFLICT OF INTEREST STATEMENT

The authors declare no financial or non‐financial competing interests.

## ETHICS STATEMENT

Not applicable.

## Supporting information

Supporting Information S1

## Data Availability

The data presented in the study will be made available by the corresponding author on request.
