# Supplementary material for: Survivin inhibition ameliorates liver fibrosis by inducing hepatic stellate cell senescence and depleting hepatic macrophage population
Source: J Cell Commun Signal. 2024 Jan 25;18(1):e12015. doi: 10.1002/ccs3.12015 (PMC10964939; doi:10.1002/ccs3.12015)
Supplement: Supplementary file 1 — Supporting Information S1 [file CCS3-18-e12015-s001.docx]

**Supplementary Information**

**Survivin inhibition ameliorates liver fibrosis by inducing hepatic stellate cell senescence and depleting hepatic macrophage population**

**Authors:** Sachin Sharma, Shaikh Maryam Ghufran, Mehreen Aftab, Chhagan Bihari, Sampa Ghose, Subhrajit Biswas*

**Contents:**

1. Methods
2. Table S1: Quantitative PCR (qPCR) human primer nucleotide sequences
3. Table S2: Quantitative PCR (qPCR) mouse primer nucleotide sequences
4. Table S3: Details of primary and secondary antibodies
5. Supplementary Figures with Legends (S1 to S6)

**Methods**

**Chemical reagents and recombinant protein**

Reagents used in the present study were as follows: MTT (3-(4,5-dimethylthiazol-2-yl)-2,5-diphenyltetrazolium bromide, (#TC191, himedia, India), YM155 (#S1130, selleckchem, USA), LY2109761 (#S2704, selleckchem, USA), dimethyl sulfoxide (DMSO) (#D8418, sigma-aldrich, USA), human TGF-β1, (#100-21, peprotech, USA).

**Data acquisition from the GEO database**

The α-SMA (ACTA2) and survivin (BIRC5) expression from GSE49541 GEO data of whole liver included METAVIR score F0/F1 vs F3/F4. The GSE68001 dataset including data from activated, quiescent, and reverted HSCs was downloaded from the publicly available National Cancer for Biotechnology Information Gene Expression Omnibus (GEO) database. The data contained different numbers of samples for each gene expression and we used the log2-transformed values for subsequent analyses.

**Human HSC cell line culture**

Human hepatic stellate cell line, LX2 was obtained from Dr. Scott L Friedman and routinely cultured in DMEM supplemented with 4 mmol/L L-glutamine, 100 IU/ml Penicillin/100 µg/ml Streptomycin, and 2 % (v/v) FBS (#RM1112, himedia, India) at 37^0^C with 5% CO2 in the incubator. Cells were passaged by Trypsin-EDTA solution (#TCL007, himedia, India) at 70-80% confluency.

**Treatment of HSC cells**

Cells were pre-treated with inhibitory drugs 45 minutes before TGF-β1 cytokine treatment in serum free media. siRNA transfection related experiments were performed after 72 hours of siRNA transfection to cells. For the conditioned media study, we incubated HSCs with 1:1 (v:v) macrophage conditioned media/fresh serum free DMEM for 24 hours.

**Induction of senescence**

HSCs were treated with 10 nM YM155 and 40 nM siRNA survivin (#4390824, thermo scientific, USA) in DMEM with 2% (v/v) FBS for 24 hours, and 48 hours respectively. Cells were washed once and then replenished with routine media. The medium was changed on the fourth day. Induction of senescence on the fifth day was confirmed by SA-β-gal assay.

**RNA isolation and quantitative polymerase chain reaction (qPCR)**

Total RNA was isolated from cells using trizol reagent (#15596026, invitrogen, USA), and cDNA synthesis was performed with RevertAid First Stand cDNA synthesis kit (#K1622, thermo scientific, USA) using random primers. Quantitative PCR was performed using sybr fluorochrome master mix (#A25742, applied biosystems, USA) on StepOnePlus PCR System (#4376600, applied biosystems, USA) using gene-specific primers (Supplementary Table S1 and S2).

**Measurement of cell viability**

Cell viability assay was performed using MTT (#TC191, himedia, India). Cells were seeded (5×10^4^ cell/well) into 96-well tissue culture plates containing 100 μL DMEM with FBS. After 24 h of incubation (37ºC, 5% CO2), the medium was carefully removed. Cells were then treated with respective concentrations of YM155, and incubated for another 24 hours. MTT with 0.5mg/ml final concentration was added to each well and incubated for 4 hours. An equal volume of DMSO was added to solubilize the formazan crystals. After 15 minutes of incubation at 37^o^C, absorbance was recorded using a multiskan multifunction microplate reader (#51119100, thermo scientific, USA) at 570 nm wavelength. All experiments were performed in triplicate.

**Migration assay**

**Wound healing scratch assay-**

Cells (0.3x10^6^ cells) were seeded in 6 well plates and grown to 100% confluence monolayer. Cells were serum starved overnight with DMEM (supplemented with 0.2% FBS). Cells were washed and a wound was produced using a p200 pipet tip in a straight line by creating a scratch. Cells were placed in a CO_2_ incubator at 37^o^C and the images were taken at 0 hours, and 24 hours of treatment, in the same field views. Based on the width of the wound, we calculated the migration distance using Nikon NIS elements advanced research software.

**Transwell migration assay-**

Cells (0.05x10^6^ cells) were seeded in the upper chambers (with 3 μm membrane porosity) of transwell plates. Cells were treated according to experimental conditions and incubated in a CO_2_ incubator at 37^o^C for 24 hours. Cells migrated to the lower chamber were stained with 0.2% (w/v) crystal violet (#C0775, sigma-aldrich, USA). Migrated cells were analyzed using bright field microscopy and spectrophotometer reading using a multiskan multifunction microplate reader at 590 nm wavelength.

**3D collagen I gel contraction assay**

A collagen suspension containing 3 mg/ml of of collagen type I (#A1048301, thermo scientific, USA) in 1X DMEM was mixed with 1 × 10^6^ HSC cells. This solution was allowed to polymerize for 1 hour at 37 °C in a 24 well plate. Once polymerized, gels were analysed for detachment from the culture plate wells. Cells containing gel were treated according to experimental conditions, and digital images were taken at different time intervals using a Nikon Coolpix digital camera (Nikon, Mississauga, ON, Canada). Measurement of collagen gel diameter at the indicated time points was performed using Image J imaging software (NIH, Bethesda, MD). Gels were measured by tracing around the edges of the gel disk and the measurements were normalized to their respective well size in each image. Gel contraction experiments were performed in duplicates in three independent experiments.

**β-galactosidase assay**

**X-gal blue assay-**

Cells were fixed with 2% formaldehyde and 0.2% glutaraldehyde in phosphate-buffered saline (PBS; #TS1119, himedia, India) for 10 minutes, followed by gentle washing with PBS. Cells were stained for 24 hours in X-gal staining solution (1 mg/mL X-gal, 40 mM citric acid/sodium phosphate [pH 6.0 for human cells and pH 5.0 for mouse cells], 5 mM potassium ferricyanide, 5 mM potassium ferrocyanide, 150 mM NaCl, 2 mM MgCl2) at 37^o^C in a non-CO2 incubator. Senescent Cells stained blue color and were identified and counted using bright field microscopy.

**Flow cytometry-based β-galactosidase assay-**

Cells were harvested and fixed with 2% formaldehyde and 0.2% glutaraldehyde in PBS, followed by gentle washing with PBS. Cells were incubated with 1:2000 dilution of cell event senescence green flow cytometry assay kit (#C10840, thermo scientific, USA) for 24 hours. After incubation, cells were washed with PBS and then analyzed on BD Accuri flow cytometer using a 488-nm laser.

**Mouse frozen liver tissue β-galactosidase assay**

Fresh liver tissue was rinsed with PBS to remove residual blood. Immediately, tissues were embedded in optimal cutting temperature (OCT) compound and frozen in liquid nitrogen containing isopentane (#19387, fisher scientific, USA). Frozen samples were stored at -80^o^C for future cryostat sectioning. 10 μm thick frozen liver tissues were sectioned using cryostat (#CM1520, leica biosystem, Germany). Liver sections were fixed with 2% formaldehyde and 0.2% glutaraldehyde in PBS for 10 minutes, followed by gentle washing with PBS. Tissue sections were stained for 24 hours in X-gal staining solution (0.5 mg/mL X-gal, 40 mM citric acid/sodium phosphate [pH 5.5], 5 mM potassium ferricyanide, 5 mM potassium ferrocyanide, 150 mM NaCl, 2 mM MgCl2) at 37^o^C in a non-CO2 incubator. The tissue sections showing blue color were identified and positive cells were counted in bright field microscopy using Leica DM IL LED (leica biosystem, Germany).

**Cell cycle assay by flow cytometry**

Cells were fixed in 70% ethanol (in PBS) for 1 hour at 4^o^C, washed and stained with 50 µg/ml propidium iodide solution containing 100 µg/ml RNase A, at 37^o^C in the dark. Data was acquired using a flow cytometer (BD Accuri; BD Biosciences, USA) and DNA content was measured at 605 nm wavelength.

**Macrophage polarization and characterization**

Human monocytic THP-1 cells were cultured in RPMI 1640 medium (#AT162, himedia, India) containing 10% FBS, and maintained at 37°C in a 5% CO2 incubator. For macrophage differentiation, THP1 cells were seeded at a density of 0.5 × 10^6^ cells/ml and treated with 50 ng/ml phorbol 12-myristate 13-acetate (PMA; #79346, sigma-aldrich, USA) for 24 hours followed by incubation in fresh PMA free complete medium for another 48 hours. These differentiated macrophages were called M0 macrophages. For M1-like polarization, we incubated M0 macrophages with lipopolysaccharide (LPS; #L4391, sigma-aldrich, USA) and INFγ (#300-02, peprotech, USA) 20 ng/ml each for 24 hours, for M2-like polarization M0 macrophages were treated with IL-4 (#200-04, peprotech, USA) and IL13 (#200-13, peprotech, USA) 20 ng/ml each. Macrophage subtypes were characterized by analyzing surface markers and cytokine gene expression by qPCR. Macrophages were cultured with RMPI with 1% (v/v) FBS for 24 hours to collect conditioned media.

**TGFβ1 cytokine ELISA**

Macrophage conditioned media was collected and the concentration of TGFβ1 cytokine in the conditioned media was measured using TGFβ1 cytokine ELISA kit (#88-8350-22, thermo scientific, USA).

**Western Blot**

Samples were homogenized in cold RIPA buffer (50 mM Tris–HCl, 150 mM NaCl, 0.1% SDS, with protease inhibitor cocktail (04693132001, sigma-aldrich, USA) and phosphatase inhibitor (#PHOSS-RO, sigma-aldrich, USA) in 10 ml), and the lysates were centrifuged to collect protein. The samples were boiled in lamelli buffer and run on SDS-PAGE gels followed by protein transfer on PVDF membrane. The membranes were developed according to the standard protocols using primary and secondary antibodies (Supplementary Table S3). The bands were visualized using an ECL detection reagent (#37074, thermo scientific, USA) by ImageQuant LAS 500 chemiluminescence. The intensity of individual bands was quantified using ImageJ densitometry software and normalized with a loading control.

**Liver Enzymes and Staining Procedures**

Blood was collected at the time of tissue excision and plasma serum was isolated. ALT and AST in the serum were measured. Formalin-fixed liver samples were embedded in paraffin and sectioned (5 μm thickness), stained with Hematoxylin (Mayers; S058, himedia, India) & Eosin Y (#230251, sigma-aldrich, USA), and sirius red (#365548, sigma-aldrich, India). The sirius red-positive area was measured at five random fields at a magnification of 20x and analyzed using National Institutes of Health Image J software. The results are presented as the percent of area positively stained for sirius red in the liver tissue sections.

**Immunofluorescence**

**Cultured cells-**

Cells were grown over coverslips and after proper attachment, they were treated according to experimental conditions. After overnight incubation, media was aspirated and 4% paraformaldehyde was added for 10 minutes at room temperature which allows to fix the cell, followed by washing twice with PBS. Cells were incubated in a blocking buffer containing 5% BSA in saline for at least 1 hr. After incubation, the blocking buffer was aspirated, and cells were incubated overnight with the primary antibody in a humid closed chamber. After incubation, coverslips were rinsed with PBS followed by incubation in fluorochrome tagged secondary antibody for 2 hours at room temperature in dark. Finally, coverslips were mounted with DAPI mounting media (#H-1200, vector laboratory, USA). Confocal images were taken using Carl Zeiss LSM 880 scanning confocal microscope and quantification was done by using Image J imaging software.

**Liver tissue sections-**

Formalin fixed paraffin embedded tissue sections (5 μm thickness) were deparaffinized in xylene and rehydrated in decreasing grades of ethanol. Tissue sections were blocked in 3% H_2_O_2_ for 10 minutes followed by antigen retrieval in 10 mM citrate buffer (pH 6). Tissue sections were blocked in 5% serum supplemented with 0.15% triton X-100 in PBS for 60 minutes. Slides were incubated with primary antibody diluted in 1% serum with 0.15% triton X-100 in PBS, overnight at 4^o^C. Following washing three times with PBS, tissue sections were incubated with fluorochrome tagged secondary antibody for 1 hour at room temperature in dark. Then the coverslips were mounted with DAPI mounting media (#H-1200, vector laboratory, USA). Carl Zeiss LSM 880 scanning confocal microscope was used to take images and quantification was done by using Image J imaging software.

**Immunohistochemistry**

Deparaffinized formalin fixed paraffin embedded tissue sections (5 μm thickness) were dehydrated in xylene and rehydrated in decreasing grades of ethanol. Tissue sections were blocked in 3% H_2_O_2_ for 20 minutes followed by antigen retrieval in 10 mM citrate buffer (pH 6). Tissue sections were stained according to the manufacturer’s instructions (#OSH001, pathnsitu biotechnologies, India). The sections were dehydrated with gradually increasing grades of ethanol and xylene followed by mounting in DPX mounting media (#44581 sigma-aldrich, USA). Sections were visualized in bright field microscopy using Leica DM IL LED (leica biosystem, Germany).

**Table S1**: Quantitative PCR (qPCR) human primer nucleotide sequences

| **Gene Symbol** | **Accession No.** | **Nucleotide Sequence (5' to 3')** | |
| --- | --- | --- | --- |
| Survivin (BRIC5) | NM_001168 | **Forward** | GTTGCGCTTTCCTTTCTGTC |
|  |  | **Reverse** | CTTTCTCCGCAGTTTCCTCA |
| α-Smooth Muscle Actin (α-SMA) | NM_001141945 | **Forward** | CAATGGCTCTGGGCTCTGTAA |
|  |  | **Reverse** | ACCATCACCCCCTGATGTCT |
| Collagen Type I α(I) chain (COL1A1) | NM_000088 | **Forward** | GACGAGACCAAGAACTGCCC |
|  |  | **Reverse** | TTGGTCGGTGGGTGACTCT |
| Fibronectin (FN1) | NM_212482 | **Forward** | TGGGCAACTCTGTCAACGAA |
|  |  | **Reverse** | CCACTCATCTCCAACGGCAT |
| Lysyl Oxidase (LOX) | NM_002317 | **Forward** | CAGATTTCTTACCCAGCCGACC |
|  |  | **Reverse** | GGCATCAAGCAGGTCATAGTGG |
| Chemokine (C-C motif) ligand 2 (CCL2) | NM_002982 | **Forward** | TCAAACTGAAGCTCGCACTCT |
|  |  | **Reverse** | GGGGCATTGATTGCATCTGGC |
| CD14 | NM_000591 | **Forward** | TGCCGCTGTGTAGGAAAGAA |
|  |  | **Reverse** | CGCGCTCCATGGTCGATA |
| CD86 | NM_001001548 | **Forward** | TTGGGAAAGTCACTGCGACA |
|  |  | **Reverse** | ACGTCGGATTCAAATACAGCA |
| CD68 | NM_001251 | **Forward** | CCTCCAAGCCCAGATTCAGA |
|  |  | **Reverse** | CACAGCTTCCCTGGACCTTG |
| CD80 | NM_005191 | **Forward** | GCAGGGAACATCACCATCCA |
|  |  | **Reverse** | ACGTGGATAACACCTGAACAGA |
| CD86 | NM_175862 | **Forward** | TCCCCCAGACCACATTCCTTG |
|  |  | **Reverse** | TCTTCCCTCTCCATTGTGTTGG |
| Tumor necrosis factor-α (TNF-α) | NM_000594 | **Forward** | CCCATGTTGTAGCAAACCCTC |
|  |  | **Reverse** | TATCTCTCAGCTCCACGCCA |
| Interleukin-1β (IL-1β) | NM_000576 | **Forward** | AGCTCGCCAGTGAAATGATG |
|  |  | **Reverse** | TCGGAGATTCGTAGCTGGATG |
| Interleukin-6 (IL-6) | NM_000600 | **Forward** | TAGTGAGGAACAAGCCAGAGC |
|  |  | **Reverse** | TGGGTCAGGGGTGGTTATTG |
| CD206 | NM_002438 | **Forward** | CACGATCCGACCCTTCCTTG |
|  |  | **Reverse** | GCTTGCAGTATGTCTCCGCT |
| CD209 | NM_001144897 | **Forward** | GAGTTCTGGACACTGGGGGA |
|  |  | **Reverse** | AAGACACCCTGCTAAGCTCT |
| Interleukin-10 (IL-10) | NM_000572 | **Forward** | AAGACCCAGACATCAAGGCG |
|  |  | **Reverse** | AATCGATGACAGCGCCGTAG |
| Transforming growth factor-β1 (TGF-β1) | NM_000660 | **Forward** | GCCGTGGAGGGGAAATTGAG |
|  |  | **Reverse** | GAACCCGTTGATGTCCACTTGC |
| 18S rRNA | NM_022551 | **Forward** | GCAATTATTCCCCATGAATG |
|  |  | **Reverse** | GGCCTCACTAAACCATCCAA |

**Table S2**: Quantitative PCR (qPCR) mouse primer nucleotide sequences

| **Gene Symbol** | **Accession No.** | **Nucleotide Sequence (5' to 3')** | |
| --- | --- | --- | --- |
| Survivin (BRIC5) | NM_009689 | **Forward** | CTCAAGAACTACCGCATCGC |
|  |  | **Reverse** | CCAAATCAGGCTCGTTCTCG |
| α-Smooth Muscle Actin (αSMA) | NM_007392 | **Forward** | GGCTCTGGGCTCTGTAAGG |
|  |  | **Reverse** | CTCTTGCTCTGGGCTTCATC |
| Collagen Type I α(I) chain (COL1A1) | NM_007742 | **Forward** | ATCAGCTGGAGTTTCCGTGC |
|  |  | **Reverse** | GGACCCATTGGACCTGAACC |
| Fibronectin (FN1) | NM_010233 | **Forward** | CGAGGTGACAGAGACCACAA |
|  |  | **Reverse** | CTGGAGTCAAGCCAGACACA |
| Lysyl Oxidase (LOX) | NM_010728 | **Forward** | GACCACAGGGTACTGCTACG |
|  |  | **Reverse** | CCATGCTGTGGTAATGTTGGTG |
| Chemokine (C-C motif) ligand 2 (CCL2) | NM_011333 | **Forward** | CACTCACCTGCTGCTACTCA |
|  |  | **Reverse** | GCTTGGTGACAAAAACTACAGC |
| Peroxisome proliferator-activated receptor γ (PPARγ) | NM_001127330 | **Forward** | GGTGTGATCTTAACTGCCGGA |
|  |  | **Reverse** | CCCAAACCTGATGGCATTGTG |
| F4/80 | NM_010130 | **Forward** | TCACCTTGTGGTCCTAACTCAG |
|  |  | **Reverse** | TCAGACACTCATCAACATCTGCG |
| Inducible nitric oxide synthase 2 (iNOS2) | NM_010927 | **Forward** | GAGACAGGGAAGTCTGAAGCAC |
|  |  | **Reverse** | CCAGCAGTAGTTGCTCCTCTTC |
| CD80 | NM_001359898 | **Forward** | GGCAAGGCAGCAATACCTTA |
|  |  | **Reverse** | CTCTTTGTGCTGCTGATTCG |
| CD86 | NM_019388 | **Forward** | CCACGATGGACCCCAGATG |
|  |  | **Reverse** | CCTTTGTAAATGGGCACGGC |
| Tumor necrosis factor-α (TNFα) | NM_013693 | **Forward** | TAGCCCACGTCGTAGCAAAC |
|  |  | **Reverse** | ACAAGGTACAACCCATCGGC |
| Interleukin-1β (IL-1β) | NM_008361 | **Forward** | TGCCACCTTTTGACAGTGATG |
|  |  | **Reverse** | TGATGTGCTGCTGCGAGATT |
| Interleukin-6 (IL-6) | NM_031168 | **Forward** | GGATACCACTCCCAACAGACC |
|  |  | **Reverse** | TTCTGCAAGTGCATCATCGT |
| CD206 | NM_008625 | **Forward** | GTTCACCTGGAGTGATGGTTCTC |
|  |  | **Reverse** | AGGACATGCCAGGGTCACCTTT |
| Arginase 1 (ARG1) | NM_007482 | **Forward** | CATTGGCTTGCGAGACGTAGAC |
|  |  | **Reverse** | GCTGAAGGTCTCTTCCATCACC |
| Chitinase 3-like 3 Protein (YM1) | NM_009892 | **Forward** | GAAGCTCTCCAGAAGCAATCC |
|  |  | **Reverse** | ATCAGCTGGTAGGAAGATCCCAG |
| Interleukin-13 (IL-13) | NM_008355 | **Forward** | CCAGGTCCACACTCCATACC |
|  |  | **Reverse** | TGCCAAGATCTGTGTCTCTCC |
| Interleukin-10 (IL-10) | NM_010548 | **Forward** | ACCTGGTAGAAGTGATGCCC |
|  |  | **Reverse** | ACAGGGGAGAAATCGATGACAG |
| Transforming growth factor-β1 (TGF-β1) | NM_011577 | **Forward** | CTGCTGACCCCCACTGATAC |
|  |  | **Reverse** | AGCCCTGTATTCCGTCTCCT |
| 18S rRNA | NR_003278 | **Forward** | GTTGGTTTTCGGAACTGAGGC |
|  |  | **Reverse** | TTTCGCTCTGGTCCGTCTTG |

**Table S3**: Primary and secondary antibodies details

| **Target Protein** | **Company** | **Application** | **Dilution** |
| --- | --- | --- | --- |
| Survivin | CST, #2808 | Immunoblot | 1:1000 |
|  |  | Immunofluorescence | 1:200 |
| α-SMA | CST, #19245 | Immunoblot | 1:1000 |
| α-SMA | Abcam, #ab7817 | Immunofluorescence | 6.82 μg/ml |
| Collagen I | CST, #72026 | Immunoblot | 1:1000 |
|  |  | Immunofluorescence |  |
| Collagen I | Abcam, #ab34710 | Immunohistochemistry | 1:100 |
| Fibronectin | Abcam, #ab2413 | Immunoblot | 1:2000 |
| pSMAD2 | CST, #3108 | Immunoblot | 1:1000 |
| SMAD2/3 | CST, #3102 | Immunoblot | 1:1000 |
| MMP2 | Abcam, #ab37150 | Immunoblot | 1:1000 |
| MMP9 | Abcam, # ab38898 | Immunoblot | 1:1000 |
| GAPDH | CST, #5174 | Immunoblot | 1:2000 |
| Α-Tubulin | CST, #2144 | Immunoblot | 1:2000 |
| iNOS | Invitrogen, #MA5-17139 | Immunohistochemistry | 1:100 |
|  |  | Immunofluorescence |  |
| Arginase 1 | Invitrogen, #MA5-85267 | Immunohistochemistry | 1:100 |
| F4/80 | Invitrogen, #MA1-91124 | Immunohistochemistry | 1:100 |
| CD206 | Invitrogen, #PA5-101657 | Immunohistochemistry | 1:100 |
| CD68 | Invitrogen, #PA5-32330 | Immunohistochemistry | 1:100 |
| CD163 | CST, #93498 | Immunohistochemistry | 1:100 |
| Chil3 (YM1) | Invitrogen, #PA5-81356 | Immunohistochemistry | 1:100 |
| P21 | CST, #2947 | Immunohistochemistry | 1:100 |
|  |  | Immunofluorescence | 1:200 |
| p-P53 | CST, # 9284 | Immunohistochemistry | 1:100 |
|  |  | Immunofluorescence | 1:200 |
| Ki67 | Abcam, #ab15580 | Immunofluorescence | 1:200 |
| Ki67 | CST, #9449 | Immunohistochemistry | 1:100 |
| Caspase3 | CST, #9662 | Immunoblot | 1:1000 |
| PARP | CST, #9542 | Immunoblot | 1:1000 |
| Annexin-V APC-conjugated | BD Bioscience, 550474 | Flow Cytometry | 5 μg/ml |
| Anti-rabbit HRP- conjugated  secondary antibody | Jackson Immuno Research, #111-035-144 | Immunoblot | 1:10,000 |
| Anti-mouse HRP- conjugated  secondary antibody | Jackson Immuno Research, #115-035-003 | Immunoblot | 1:10,000 |
| Anti-rabbit FITC­- conjugated secondary antibody | Abcam, #ab6717 | Immunofluorescence | 1:250 |
| Anti-mouse Alexa Fluor 647­- conjugated secondary antibody | Abcam, #ab150115 | Immunofluorescence | 1:500 |

**Supplementary Figures with Legends**


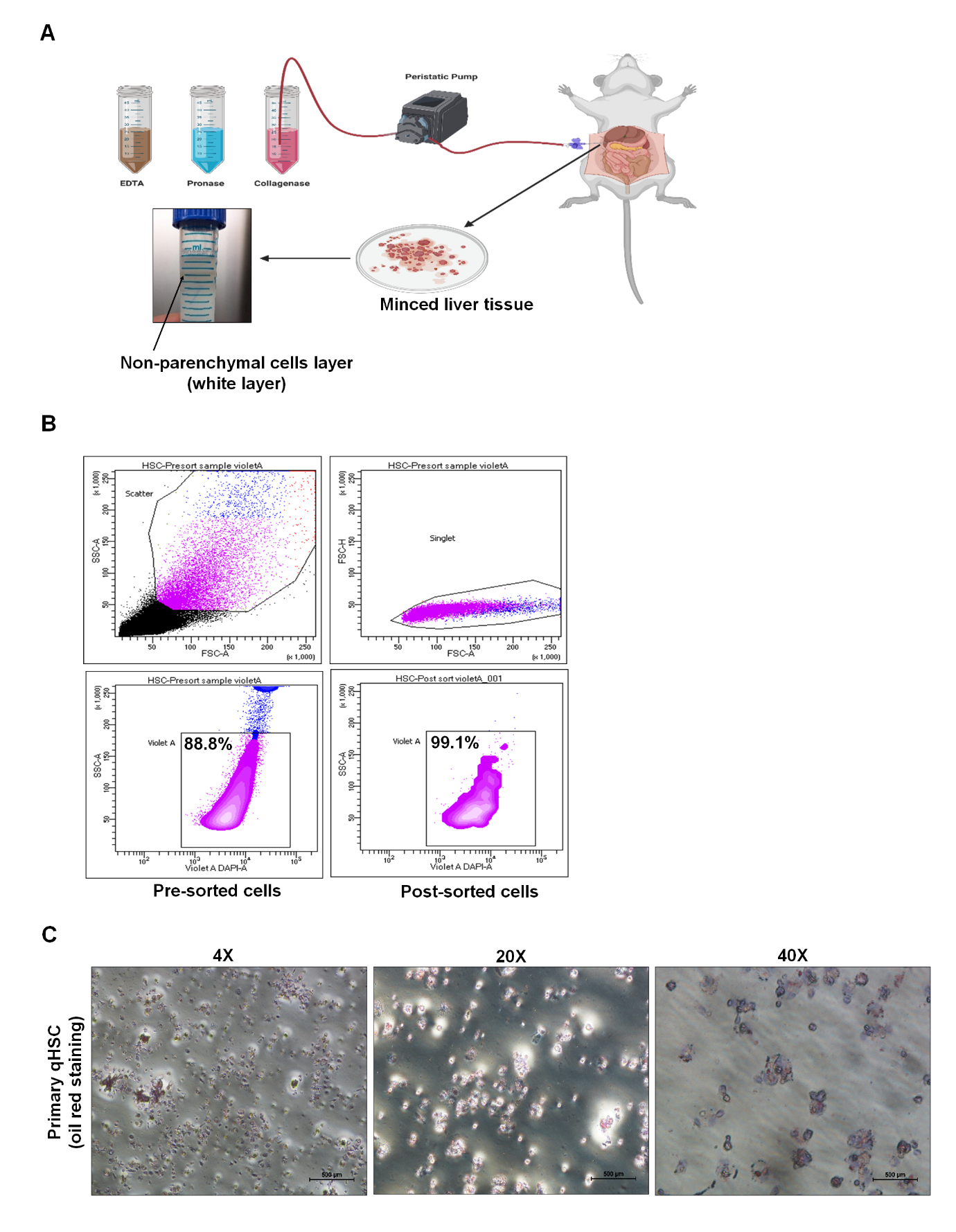


**Supplementary Figure S1.** Mouse primary hepatic stellate cell isolation and characterization. (**A**) A schematic diagram of enzymatic-liver perfusion method to isolate mouse primary hepatic stellate cells. (**B**) Retinoid-based FACS sorting improves the purity of isolated HSCs using a 405–407 nm laser for excitation and a 45+0/50 nm band-pass filter for detection. (**C**) Oil red staining of freshly isolated mouse primary HSCs.


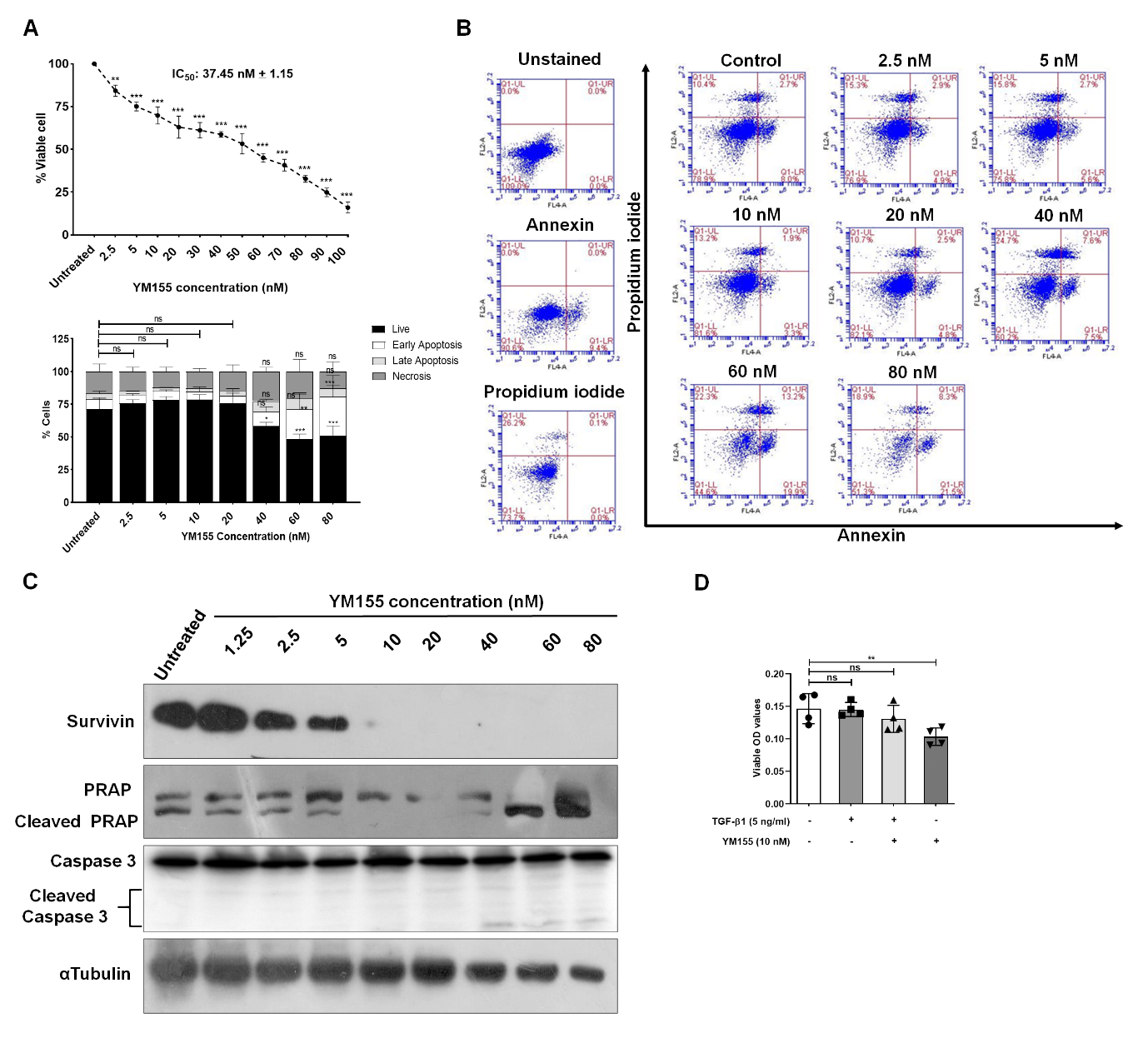


**Supplementary Figure S2.** YM155 mediated survivin inhibition decreases HSC viability dose dependently. (**A**) MTT based viability assay of LX2 cells with various YM155 concentrations after 24 hours of treatment. IC50 value calculated using non-linear regression. (**B**) Annexin-PI flow cytometry based cell death analysis of LX2 cells treated with various YM155 concentrations for 24 hours. (**C**) Western blot of LX2 cells treated with various concentrations of YM155 at 24 hours. Expression of survivin, caspase 3, PARP, and α-tubulin was evaluated. (**D**) Cell viability of LX2 cells after the treatment of TGF-β1 (5 ng/ml) and YM155 (10 nM) for 24 hours. nsP>0.05, *P<0.05, **P<0.01, ***P<0.001 [one-way ANOVA for (A, D); two-way ANOVA for (B)].

**Supplementary Figure S3.** siRNA mediated knockdown of survivin expression does not induce cell death. Flow cytometry-based cell death assay of human HSCs, LX2 cells treated with siRNA survivin (40 nM).


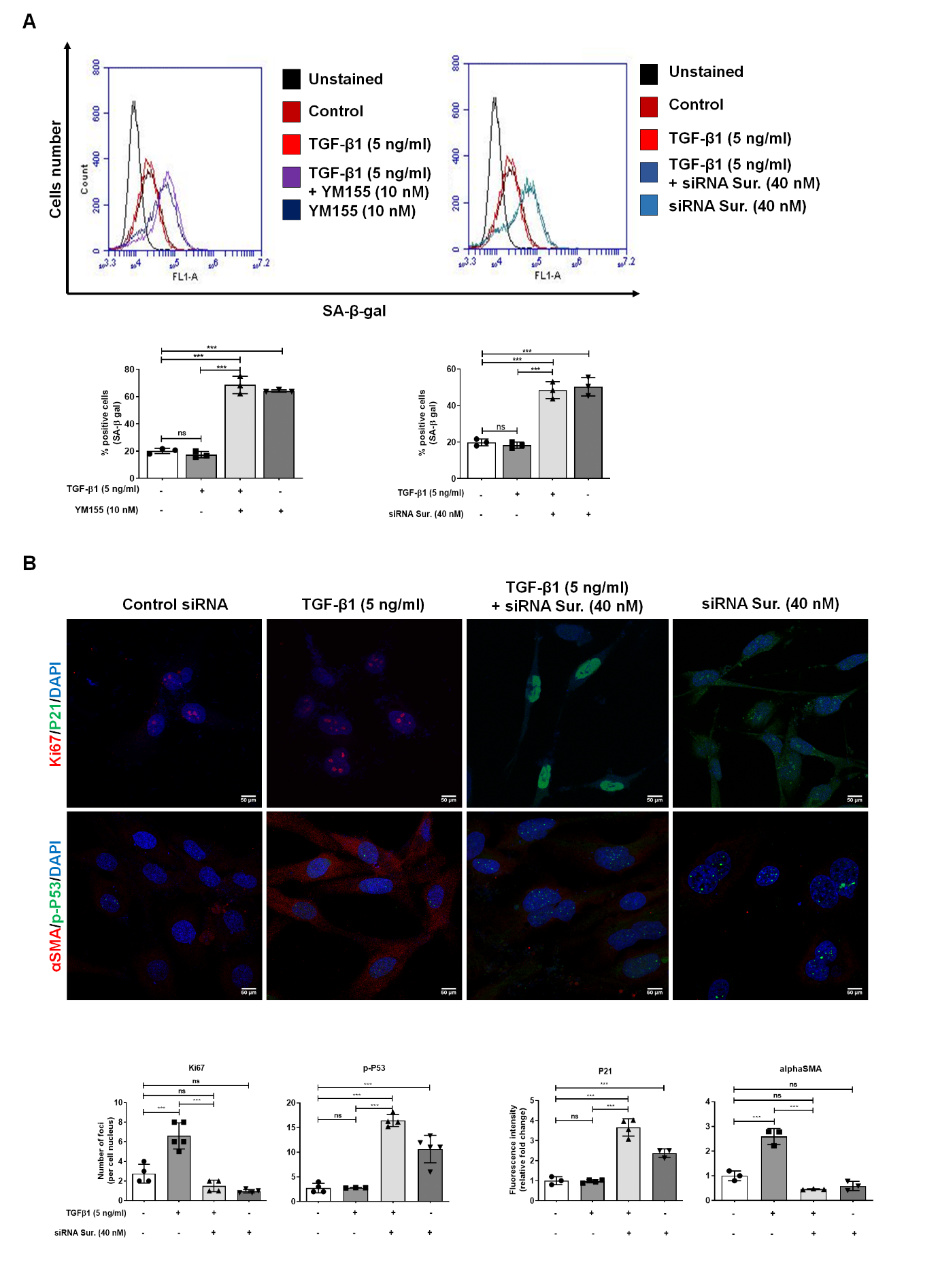


**Supplementary Figure S4.** siRNA mediated survivin expression knockdown induces cellular senescence in activated HSCs**.** (**A**) Flow cytometry based β-galactosidase (β-SA-gal) senescence assay of TGF-β1 (5 ng/ml) stimulated and TGF-β1 stimulated and YM155 (10 nM) or siRNA survivin (40 nM) treated LX2 cells. (**B**) IF images of senescent LX2 cells showing Ki67 (red), P21 (green), α-SMA (red), p-P53 (green), and nucleus (blue) in TGF-β1 stimulated, and TGF-β1 and siRNA survivin (40 nM) treated LX2 cells (scale bar: 50 μm, 63x magnification). nsP>0.05, *P<0.05, **P<0.01, ***P<0.001 [one-way ANOVA for (a, b)].


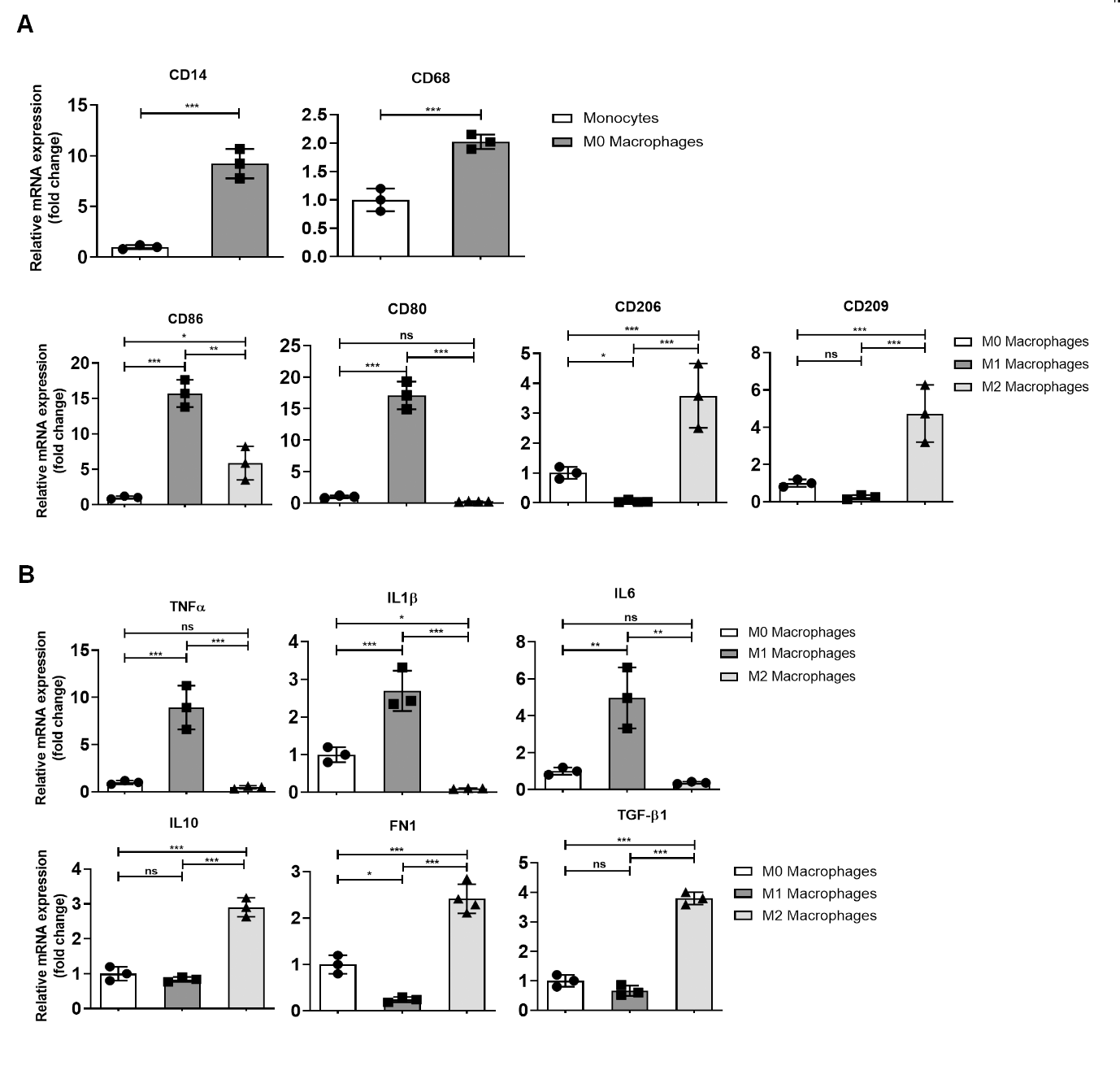


**Supplementary Figure S5.** Polarization and characterization of macrophage. (**A**) Relative mRNA expression of macrophage surface markers representing CD14, CD68 for M0 non-polarized macrophages, CD80, CD86 for M1 polarized macrophages, and CD206, CD209 for M2 polarized macrophages. (**B**) Characterization of non-polarized and polarized macrophage by analysis of relative mRNA expression of cytokine gene expression through quantitative PCR. nsP>0.05, *P<0.05, **P<0.01, ***P<0.001 [one-way ANOVA for (a, b)].


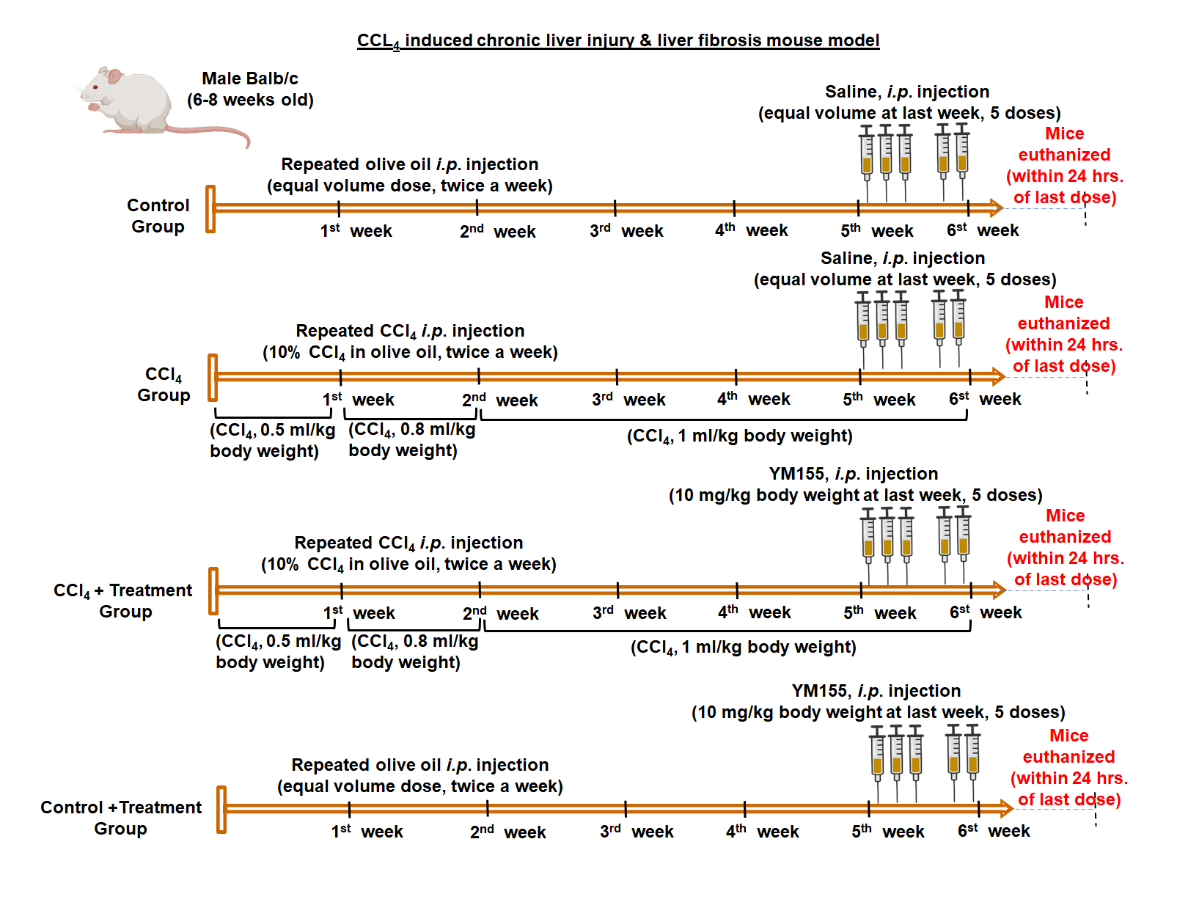


**Supplementary Figure S6.** Detailed schematic diagram of the development of liver fibrosis in male Balb/c mice. Mice were segregated into the following groups: i) Control olive oil group, ii) Fibrotic carbon tetrachloride (CCl_4_) group, iii) Fibrotic carbon tetrachloride (CCl_4_) group treated with survivin inhibitor YM155; CCl_4_+YM155, and iv) Control olive oil group treated with YM155; control+YM155. Each group consists of n=7 mice. To develop chronic injury and fibrosis, fibrotic group mice (n = 7) were administered with increasing concentrations of CCl_4_ (week 1: 0.5 ml/kg; week 2: 0.8 ml/kg and week 3–6: 1 ml/kg prepared in olive oil) twice weekly by intraperitoneal (*i.p*) injections for 6 weeks. An equal volume of olive oil was given parallel to mice as vehicle controls. During the 5^th^-week, mice were treated with five doses of 10 mg/kg of survivin inhibitor, YM155. The mice were sacrificed within 24 hours of the last dose given in 6^th^ week of CCL_4_ injections. The liver was excised and blood was collected for subsequent analyses.
